# Genomic and Phenotypic Characterization of *Chloracidobacterium* Isolates Provides Evidence for Multiple Species

**DOI:** 10.3389/fmicb.2021.704168

**Published:** 2021-06-17

**Authors:** Mohit Kumar Saini, Aswathy Sebastian, Yoshiki Shirotori, Nathan T. Soulier, Amaya M. Garcia Costas, Daniela I. Drautz-Moses, Stephan C. Schuster, Istvan Albert, Shin Haruta, Satoshi Hanada, Vera Thiel, Marcus Tank, Donald A. Bryant

**Affiliations:** ^1^Department of Biological Sciences, Tokyo Metropolitan University, Hachioji, Japan; ^2^The Huck Institutes for the Life Sciences, The Pennsylvania State University, University Park, PA, United States; ^3^Department of Biochemistry and Molecular Biology, The Pennsylvania State University, University Park, PA, United States; ^4^Department of Biology, Colorado State University-Pueblo, Pueblo, CO, United States; ^5^Singapore Centre for Environmental Life Sciences Engineering, Nanyang Technological University, Singapore, Singapore; ^6^DSMZ – German Culture Collection of Microorganisms and Cell Cultures, GmbH, Braunschweig, Germany

**Keywords:** *Acidobacteriota*, *Chloracidobacterium*, thermophile, chlorophototrophy, genome

## Abstract

*Chloracidobacterium* is the first and until now the sole genus in the phylum *Acidobacteriota* (formerly *Acidobacteria*) whose members perform chlorophyll-dependent phototrophy (i.e., chlorophototrophy). An axenic isolate of *Chloracidobacterium thermophilum* (strain B^*T*^) was previously obtained by using the inferred genome sequence from an enrichment culture and diel metatranscriptomic profiling analyses *in situ* to direct adjustments to the growth medium and incubation conditions, and thereby a defined growth medium for *Chloracidobacterium thermophilum* was developed. These advances allowed eight additional strains of *Chloracidobacterium* spp. to be isolated from microbial mat samples collected from Mushroom Spring, Yellowstone National Park, United States, at temperatures of 41, 52, and 60°C; an axenic strain was also isolated from Rupite hot spring in Bulgaria. All isolates are obligately photoheterotrophic, microaerophilic, non-motile, thermophilic, rod-shaped bacteria. *Chloracidobacterium* spp. synthesize multiple types of (bacterio-)chlorophylls and have type-1 reaction centers like those of green sulfur bacteria. Light harvesting is accomplished by the bacteriochlorophyll *a*-binding, Fenna-Matthews-Olson protein and chlorosomes containing bacteriochlorophyll *c*. Their genomes are approximately 3.7 Mbp in size and comprise two circular chromosomes with sizes of approximately 2.7 Mbp and 1.0 Mbp. Comparative genomic studies and phenotypic properties indicate that the nine isolates represent three species within the genus *Chloracidobacterium*. In addition to *C. thermophilum*, the microbial mats at Mushroom Spring contain a second species, tentatively named *Chloracidobacterium aggregatum*, which grows as aggregates in liquid cultures. The Bulgarian isolate, tentatively named *Chloracidobacterium validum*, will be proposed as the type species of the genus, *Chloracidobacterium.* Additionally, *Chloracidobacterium* will be proposed as the type genus of a new family, *Chloracidobacteriaceae*, within the order *Blastocatellales*, the class *Blastocatellia*, and the phylum *Acidobacteriota.*

## Introduction

Bacteria in the phylum *Acidobacteria* were first reported in 1997 (Kuske et al., 1997; Ludwig et al., 1997); the phylum was validly described in 2012 (Euzeby, 2012) and recently renamed *Acidobacteriota* (Oren et al., 2015; Whitman et al., 2018). In the *Acidobacteriota*, 26 subdivisions have been proposed based on 16S rRNA gene phylogeny by culture-independent analyses. However, only 57 cultivable species belonging to 28 genera have been reported to date; the isolates belong to only seven of the 26 subdivisions (subdivisions 1, 3, 4, 6, 8, 10, and 23) (Foesel et al., 2013; Dedysh and Yilmaz, 2018; Oshkin et al., 2019). The genus *Chloracidobacterium* (domain Bacteria, phylum *Acidobacteriota*, class *Blastocatellia*, order *Blastocatellales*, proposed family *Chloracidobacteriaceae*) is presently the only genus within the phylum *Acidobacteriota* that contains members that perform chlorophototrophy [i.e., chlorophyll (Chl)-dependent phototrophy]. Originally discovered through bioinformatic analyses of metagenomic data from microbial mats associated with Octopus and Mushroom Springs in Yellowstone National Park (YNP), WY, United States, an initial enrichment culture containing “*Candidatus* (*Ca*.) Chloracidobacterium (C.) thermophilum” and growing on a defined medium was first described in 2007 (Bryant et al., 2007). This enrichment culture initially contained the cyanobacterium *Synechococcus* sp. JA-2-3B′ a(2-13) (Bhaya et al., 2007; Thiel et al., 2018), *Anoxybacillus ayderensis* (Thiel et al., 2017), and *Meiothermus ruber* (Thiel et al., 2015). Elimination of the cyanobacterium led to an enrichment culture containing “*Ca.* C. thermophilum” as the only chlorophototroph (Bryant et al., 2007). Using clues obtained from the consensus genome sequence of the non-clonal enrichment culture (Garcia Costas et al., 2012a) and diel transcription profiles for the major organisms found in the mats of Mushroom Spring *in situ* (Liu et al., 2011, 2012), the growth medium was gradually amended and improved. This culminated in the establishment of an axenic culture of *C. thermophilum* strain B^*T*^ (2015) that grows well on a completely defined medium (Tank and Bryant, 2015a, b; Tank et al., 2018). Because of the limited number of axenic isolates, the higher-level taxonomic position (i.e., family and order) for this bacterium has not been validly described to present. However, it has been assumed that *C. thermophilum* strain B^*T*^ (2015) potentially represents a novel family and perhaps a novel order (Dedysh and Yilmaz, 2018).

*Chloracidobacterium thermophilum* strain B^*T*^ (2015) employs chlorosomes containing bacteriochlorophyll (BChl) *c* and the BChl *a*-binding, Fenna-Matthews-Olson protein (FMO) for light harvesting (Tsukatani et al., 2010; Wen et al., 2011; Garcia Costas et al., 2011, 2012b). This bacterium has homodimeric type-1 reaction centers containing Chl *a*, BChl *a*, and Zn-BChl *a*′ for light energy transduction (Tsukatani et al., 2012; He et al., 2019). The primary donor of the reaction centers (i.e., the special pair, P840) comprises two Zn-BChl *a*′ molecules (Charles et al., 2020), and Chl *a* is the primary electron acceptor in these unusual type-1 reaction centers (Zill et al., 2018). *Chloracidobacterium thermophilum* strain B^*T*^ (2015) is a chlorophotoheterotroph that requires all three branched-chain amino acids, lysine, vitamin B_12_, and a reduced sulfur source (Tank and Bryant, 2015a, b). This strain is unable to grow at air-levels of oxygen, but it nevertheless obligately requires oxygen to make tyrosine and (B)Chls; thus, it is a microaerophile (Garcia Costas et al., 2012a; Tank and Bryant, 2015b). Considering that the organism has a photosynthetic apparatus that is very similar to that of the strictly anaerobic green sulfur bacteria, the oxygen relations of *C. thermophilum* are surprising. *Chloracidobacterium thermophilum* strain B^*T*^ (2015) can metabolize 18 of the 20 common amino acids (all except aspartic and glutamic acids), and it uses these compounds as its primary sources of nitrogen and reduced carbon (Tank and Bryant, 2015b, Tank et al., 2018). Growth is stimulated by bicarbonate, possibly because of the elevated growth temperature of most strains (∼50°C) and because of anaplerotic carbon fixation by carboxylation of succinyl CoA to produce 2-oxoglutarate, the immediate precursor of Chls and BChls (Tank and Bryant, 2015b).

Previous surveys of mats associated with various thermal features in YNP suggested the occurrence of different species or ecotypes (or both) of *Chloracidobacterium* spp. (Miller et al., 2009; Garcia Costas, 2010; Ross et al., 2012). Miller et al. (2009) reported evidence for four similar but distinctive types of *Chloracidobacterium*-like organisms along temperature gradients (∼35 to 75°C) associated with White Creek and Rabbit Creek in YNP. A closely related organism occurs in the Ojo Caliente hot springs in New Mexico, United States (Hallenbeck et al., 2016). Related organisms have also been detected in surveys of microbial mats associated with hot springs in Tibet (Yim et al., 2006; Lau et al., 2009), Thailand (Kanokratana et al., 2004), and Japan (Ward et al., 2017). These survey studies provide strong evidence that different species and/or ecotypes of *Chloracidobacterium* are associated with the microbial mats of alkaline and circum-neutral hot springs world-wide (Thiel et al., 2018).

An improved understanding of the ecophysiology and distinctive metabolism of *C. thermophilum*, as well as the development of a defined medium for its cultivation, provided an opportunity to attempt the isolation of additional strains of *Chloracidobacterium* across the temperature range from about 40 to 60°C at Mushroom Spring. Additionally, a sampling expedition to Rupite hot springs in Bulgaria in 2015 (Strunecký et al., 2018) provided an opportunity to isolate an axenic strain from another continent. To determine the relationships among these strains, the genomes of eight new axenic isolates were completely sequenced. Furthermore, the genome of the clonal axenic isolate [*C. thermophilum* strain B^*T*^ (2015)] derived from the original enrichment culture that had been sequenced by pyrosequencing (Garcia Costas et al., 2012a) was resequenced using the PacBio RS-II platform. Comparative analyses of these data suggest that these nine strains represent at least three species of *Chloracidobacterium*. The eight isolates from Mushroom Spring, representing two species, are more similar than any of those isolates are to the isolate from Rupite hot spring in Bulgaria.

## Materials and Methods

### *Chloracidobacterium* spp. Strains

Ten genomes from nine *Chloracidobacterium* spp. strains [strains B (2011); B^*T*^ (2015), D, 2, A, E, N, S, MS 40/45, and BV2-C] were compared in this study (Table 1). Strains D, MS40/45 and BV2-C were specifically enriched and isolated as a part of this study. Strains B^*T*^ (2015), A, 2, S, E, and N were isolated and purified previously (Tank and Bryant, 2015b; Tank et al., 2017, 2018). The current type-strain, *Chloracidobacterium thermophilum* strain B^*T*^ (2015), hereafter usually denoted simply as strain B^*T*^ (2015), is a clonal isolate derived from an enrichment culture of *Synechococcus* sp. Type B′ (Bryant et al., 2007), which was isolated from Octopus Spring (Tank and Bryant, 2015a, b; Tank et al., 2018). Octopus Spring is nearby and chemically similar to Mushroom Spring in the Lower Geyser Basin of YNP (Tank et al., 2017). For convenience, an intermediate enrichment culture, derived from the *Synechococcus* sp. enrichment, but with the cyanobacterium eliminated, will hereafter be referred to as “strain B (2011),” because the published complete genome of this non-clonal strain was used as the basis for comparing the other nine genomes described herein (Garcia Costas et al., 2012a). The non-axenic, non-clonal “strain B (2011)” was sequenced using the Roche/454 platform in 2011; the current type strain, axenic clonal isolate strain B^*T*^ (2015) and the other eight genomes described herein, were sequenced using either the PacBio RSII or Sequel platforms (Table 1).

**TABLE 1:** General information concerning ten strains of *Chloracidobacterium* spp.

Characteristics	Strain B (2011)	Strain B^*T*^ (2015)	Strain D	Strain 2	Strain A	Strain S	Strain N	Strain E	Strain MS40/45	Strain BV2-C
Habitat	Hot spring microbial mat	Hot spring microbial mat	Hot spring microbial mat	Hot spring microbial mat	Hot spring microbial mat	Hot spring microbial mal	Hot spring microbial mal	Hot spring microbial mat	Hot spring microbial mat	Hot spring microbial mat
Isolation site^a^	Octopus Spring, YNP	Octopus Spring, YNP	Mushroom Spring, YNP	Mushroom Spring, YNP	Mushroom Spring, YNP	Mushroom Spring, YNP	Mushroom Spring, YNP	Mushroom Spring, YNP	Mushroom Spring, YNP	Rupite Hot Springs, Bulgaria
Temperature (°C)	51–61	52–61	52	52	52	52	60	52	40–45	∼40
Cell suspension	Homogenous	Homogenous	Homogenous	Clumps and aggregates	Clumps and aggregates	Clumps and aggregates	Clumps and aggregates	Clumps and aggregates	Homogenous and aggregates	Aggregates
Sequencing platform	Roche 454	PacBio RSII	PacBio Sequel	PacBio RSII	PacBio RSII	PacBio RSII	PacBio Sequel	PacBio RSII	PacBio Sequel	PacBio Sequel
Genome size (bp)	3,695,372	3,756,354	3,635,588	3,766,674	3,769,663	3,757,470	3,715,176	3,777,155	3,662,232	3,659,143
Number of chromosomes	2	2	2	2	2	2	2	2	2	2
Number of contigs	2	2	2	2	2	2	2	2	2	2
G + C mol%	61.34	61.31	61.48	62.15	62.14	62.13	62.15	62.18	62.68	59.9
CDS	3218	3424	3161	3339	3411	3407	3217	3325	3097	3163
RNA genes	49	51	49	50	50	51	50	52	49	50
tRNA	46	48	46	47	47	48	47	49	46	47
rRNA operons	1	1	1	1	1	1	1	1	1	1

*^a^Octopus and Mushroom Spring are thermal features in the Lower Geyser Basin in Yellowstone National Park, WY, United States.*

Strain B was isolated from Octopus Spring, Lower Geyser Basin, YNP (GPS coordinates: Lat.: 44.53408, Long.: –110.7979), while strains D, 2, A, S, N, E, and MS 40/45 were isolated from Mushroom Spring, Lower Geyser Basin, YNP (GPS coordinates: Lat.: 44.5387, Long.: –110.798). Strain BV2-C was isolated from Pool 2, sampling site C, of the Baba Vanga Sanctuary portion of Rupite hot springs, Bulgaria (GPS coordinates: 41° 21′ N, 23° 14′ E) (see Strunecký et al., 2018). The local temperatures of the isolation sites for all of the strains are given in Table 1. Six of the strains were compared in greater detail to ascertain physiological differences among strains B^*T*^ (2015), D, N, E, MS40/45, and BV2-C.

### *Chloracidobacterium* spp. Strain Isolation

To obtain additional axenic cultures of *Chloracidobacterium* spp. (strain D, MS40/45 and BV2-C), procedures similar to those previously described were employed (Tank and Bryant, 2015b). For purification, CTM medium supplemented with washed Bacto-Agar [1% (w/v)] (Thermo Fisher Scientific, Waltham, MA) was prepared. Simultaneously, a dilution series (10^0^ – 10^–4^) was made with sterilized water from a small amount of inoculum (100 – 200 μl) taken from enriched cultures of the respective strains. Diluted mat samples were mixed with tempered agar medium and poured into Petri dishes; approximately 80 – 100 ml medium was poured into 100 × 25 mm deep Petri dishes. After cooling, the plates were placed inside transparent, air-tight, sealed plastic bags, and the bags were purged with a mixture of gases [N_2_ 80%: H_2_ 10%: CO_2_ 10% (v/v)] for 15–20 min. Inoculated agar plates were then incubated under continuous illumination from a tungsten light (60 W, 20 to 50 μmol photons m^–2^ s^–1^) at 50°C (strain D) or 45°C (strains MS40/45 and BV2-C). After incubation for three days, the bags were opened for 30 to 60 s to replace the gas phase with air so that microoxic conditions were created in the agar plates, conditions which are favorable for the growth of *Chloracidobacterium* spp. Agar plates were further incubated under the same conditions for 7 to 10 days and checked for the growth of *Chloracidobacterium* spp., which form greenish-brown, lentiform-shaped colonies embedded within the agar. Single, well-separated colonies were aseptically picked under a stereoscopic microscope (Olympus SZX7) using autoclaved Pasteur pipettes; isolates were repeatedly transferred and streaked onto fresh CTM medium until axenicity was achieved. The oxygen concentration for surface-inoculated plates was kept at about 1% (v/v) oxygen to provide the microoxic conditions necessary for growth of the strains (no isolate could grow under air-levels of oxygen). Picked colonies were checked for purity under the microscope (Nikon Eclipse E600, Nikon, Japan). Once axenicity was achieved, colonies were transferred back into freshly prepared liquid CTM medium to obtain larger amounts of bacterial biomass. For long term preservation of the isolated strains, actively growing cells were frozen at –80°C in 30% (v/v) glycerol.

### Physiological Experiments (Growth Behavior, Microscopic Examination, Pigment Analysis, and Temperature Dependence)

Bacterial strains of *Chloracidobacterium* spp., strain B^*T*^, N, and E [revived from glycerol stocks (–80°C)] as well as strains D, MS40/45 and BV2-C, were grown photoheterotrophically in freshly prepared CTM medium pH 7.5–8 for physiological studies. Cells were inoculated into 80 ml of medium in 100 ml Erlenmeyer flasks, and cultures were incubated at 50°C [B^*T*^ (2015), D, N, and E] or 45°C (MS40/45 and BV2-C) under continuous illumination from a 60W tungsten light (∼20 to 50 μmol photons m^–2^ s^–1^). To maintain a microoxic environment, flasks were incubated without shaking for the first few days until slight growth could be observed at the bottom of the flask (3 to 5 days). After initial growth had been observed, cultures were gently shaken for a few seconds by hand on alternate days. Additionally, the medium was supplemented with a mixture of the 20 proteinogenic amino acids at a concentration of 300 to 500 mg L^–1^ on day 3 or 4 to obtain higher bacterial biomass.

Growth temperature profiles were determined during growth for nine days in the temperature range 25°C to 60°C. Bacterial growth was monitored as described (Tank and Bryant, 2015b) by measuring the absorbance of BChl *c* at 667 nm in a UV-1800 UV-Vis spectrophotometer (Shimadzu, Japan). The samples for the BChl *c* measurement were prepared from 1 ml of the growing culture. Cells were centrifuged at ∼ 15,000 × *g* for 4 min and the supernatant was discarded. Pigments (BChl *c*) were extracted from the cells by resuspending samples in an acetone/methanol (7:2, v/v) solvent mixture followed by incubation in dark for 5 min. Prior to the absorbance measurements, cell debris was removed by centrifugation for 2 min at 13,000 × *g*.

Cells of all isolates were microscopically examined for their morphological features including cell shape, cell size (diameter, length), and BChl *c* fluorescence. Cells were harvested from mid-exponential growth phase, usually on day 4 after inoculation into fresh medium, and examined under the epifluorescence microscope [Eclipse E600 (NIS-Elements software D), Nikon, Japan; Xenon power supply XPS-100; monochromatic CMOS camera (Orcaflash 4.0, Hamamatsu, Japan); Filterset Ex: 350–510 nm/DM, 665 nm/Em, 830 nm LP (Semrock Inc., Rochester, NY)].

Extracted pigments were analyzed and identified by reversed-phase high performance liquid chromatography as described (Saini et al., 2020). Pigments were extracted with acetone–methanol (7:2, v/v) before filtration with a 0.2-μm polytetrafluoroethylene 4 mm, single-use filter device (Whatman) prior to injection into the column. The instrument and solvent conditions were previously described (Frigaard et al., 1997; Vogl et al., 2012).

### Genomic DNA Isolation From Strains D, N, MS40/45 and BV2-C

Strains D, N, MS40/45 and BV2-C were grown in bottles (500 ml, GL-45 screw cap bottles) containing 350 ml freshly prepared CTM medium. Each bottle was inoculated with the respective strains from mid-exponential phase cells from a primary starter culture using a 2% (v/v) inoculum. Bottles were placed into the light of a tungsten lamp (60W, 20 to 50 μmol photons m^–2^ s^–1^) and incubated at 50°C (strain D and N) and 45°C (strain MS40/45 and BV2-C) under microoxic conditions (without shaking). The medium was supplemented with a mixture of 20 proteinogenic amino acids at a concentration of 300 to 500 mg L^–1^ on day 3 or 4 to obtain higher bacterial biomass. For genomic DNA isolation, cells from late exponential phase (day 8) were harvested by centrifugation at ∼8000 × *g* for 10 min at 4°C and were washed three times with autoclaved distilled water. Total genomic DNA was extracted from the harvested cells using the Qiagen Genomic-tip 100/G kit according to the protocol of the manufacturer and eluted with elution buffer (1 ml; provided in the kit). The quantity of the extracted genomic DNA was assessed using the dsDNA Broad range (BR) assay (Life Technologies Inc., Grand Island, NY, United States) and a Qubit 3.0 Fluorometer (Invitrogen; Carlsbad, CA). The quality and purity of the extracted genomic DNA were also examined by measuring absorbance at 260 nm (A_260_) and 280 nm (A_280_) with a UV spectrophotometer (Biospec-nano, Shimadzu Biotech, Kyoto, Japan). An absorbance quotient value (A_260_/A_280_) of 1.8 < ratio (R) < 2.0 was considered to represent sufficiently purified DNA. The integrity of genomic DNA was tested by resolving DNA extracts (1 μl) on a 0.8% (w/v) agarose gel by electrophoresis (Mupid^®^ -2plus submarine electrophoresis system, Mupid Co., Ltd, Tokyo, Japan), followed by staining with GelGreen (Biotium, Hayward, CA, United States) and visualization on an LED transilluminator (Wako, Osaka, Japan). The markers used were the ExcelBand 1 Kb (0.25–10 Kb) DNA ladder (Smobio Technology, Tokyo, Japan) and the DNA loading dye was 6 × loading buffer triple dye (Nippon Gene, Wako, Osaka, Japan). Purified DNA samples were stored at –80°C until required for DNA sequence analysis.

### Genome Sequencing and Assembly

High-molecular-weight genomic DNA from strains B^*T*^ (2015), 2, A, E, and S was prepared for sequencing on the RS-II platform according to recommendations of Pacific Biosciences (Menlo Park, CA). The integrity of the genomic DNA was assessed by subjecting each DNA sample (150 ng) to electrophoresis on a 0.6% (w/v) agarose pulsed-field gel for 16 h. Sample concentrations were determined spectrophotometrically (Nanodrop; Thermo Fisher Scientific, Waltham, MA) as well as by fluorescence (Promega QuantiFluor dsDNA assay; Madison, WI). DNA (10 μg) was sheared to a size range of 10–40 kb using a Covaris g-TUBE and purified with 0.45X AMPure PB beads (Pacific Biosciences) according to the recommendations of the manufacturer. Library preparation was performed by following the 20-kb SMRTbell Template Preparation Protocol of Pacific Biosciences, using 5 μg of the purified, sheared DNA as the input. After library preparation, the libraries were assessed on an Agilent DNA 12000 bioanalyzer chip to determine the optimal cut-off for size selection. The libraries were then size-selected on a Sage Science Blue Pippin instrument, using a dye-free 0.75% agarose cassette and either 8-kb (strain S) or 15-kb [strains B^*T*^ (2015), E, A, and 2] as the cut-off and sequenced in a single SMRTcell each on a Pacific Biosciences RSII single-molecule sequencing platform at a loading concentration of 0.15 nM. The resulting sequencing reads were assembled with the Hierarchical Genome Assembly Process (HGAP) version 3 (Chin et al., 2013) as part of the SMRT Analysis 2.3.0 package^1^.

High-molecular-weight genomic DNA samples from strains D, N, MS40/45, and BV2-C were sequenced on the PacBio Sequel platform in the Genomics core facility of The Huck Institutes for the Life Sciences at The Pennsylvania State University, University Park, PA, United States. Genome assembly was performed using the Canu 1.8 assembler (Koren et al., 2017) and was circularized by Circlator 1.5.5 (Hunt et al., 2015). The complete genome sequence of the non-clonal enrichment containing strain B (2011), which has been published (Garcia Costas et al., 2012a) and is available in GenBank (Supplementary Table 1; NCBI accession number: NC_016024), was used as the reference genome for all comparative studies described herein.

### Genome Annotation and Comparison

Prior to annotation, the completeness and contamination of the genomes was checked using the online version of CheckM implemented in the Kbase software and data platform (Parks et al., 2015). In order to have a similar annotation for comparative genomics, all ten assembled genomes were annotated with the RAST annotation system (Rapid Annotation using SEED Technology) to predict the number of coding genes, total RNA genes and putative protein coding sequences (Aziz et al., 2008; Overbeek et al., 2014; Brettin et al., 2015). Complete genome sequences of all strains were submitted to NCBI under the BioProject number PRJNA717397. The NCBI accession numbers and BioSample numbers assigned to each strain are listed in Supplementary Table 1. Some comparative analyses of gene content were performed after reannotation of the genomic data by GenBank. Genomes of these strains were analyzed for their functional annotation of genes using KEGG Automatic Annotation Server (KAAS), RAST and NCBI Prokaryotic Genome Annotation Pipeline (PGAP). Thus, sequence features may vary slightly because the different pipelines use different parameters to identify coding sequences and stable RNAs. To analyze the genome organization, synteny, and rearrangements for the ten sequences genomes, multiple sequence alignments were performed using MAUVE (Darling et al., 2004). BRIG 0.95 was used to build the circular representation (Alikhan et al., 2011). Mapping studies were done using BLASTn with a cut-off value of 1 × e^–5^.

### Single-Value Whole Genome Comparisons

Single-value whole genome comparisons were calculated by computing values that summarize the similarity or distance between genomes using several indices useful for species delineation. Thus, the digital DNA-DNA hybridization (dDDH) was estimated *in silico* with the Genome-to-Genome Distance Calculator (GGDC 2.1) using the BLAST method and recommended formula 2.1 (Meier-Kolthoff et al., 2013a). The average nucleotide identities according to BLAST (ANIb), as well as the tetranucleotide values, were calculated in JspeciesWS (Richter et al., 2016). Pairwise nucleotide sequence similarity values for 16S rRNA genes were calculated with the robust global sequence alignment algorithms in the EzTaxon server^2^.

### Phylogenetic Analyses Using 16S rRNA and Whole Genome Sequences

The complete genome sequences of all ten strains were uploaded to the Type Strain Genome Server (TYGS)^3^ (Meier-Kolthoff and Göker, 2019), a free bioinformatics pipeline that can be used to perform phylogenetic analyses based upon 16S rRNA sequences or upon whole genomes for the delineation of species and subspecies. The genome sequences of other reference strains were retrieved from the comprehensive TYGS database (see text footnote 3) (Meier-Kolthoff and Göker, 2019). For phylogenomic inference, all pairwise comparisons among the complete set of genomes were calculated using genome-based distance phylogeny, and accurate intergenomic distances were inferred under the algorithm “trimming” and distance formula d5 (Meier-Kolthoff et al., 2013a); 100 distance replicates were calculated for each comparison. Digital DNA-DNA hybridization (dDDH) values and confidence intervals were calculated using the recommended settings of the genome-to-genome distance calculator version 2.1 (Meier-Kolthoff et al., 2013a). The resulting intergenomic distances were used to infer a balanced, minimum evolution tree with branch support via FASTME 2.1.4 including SPR postprocessing (Lefort et al., 2015). Branch support was inferred from 100 pseudo-bootstrap replicates each. The resulting trees were rooted at the midpoint (Farris, 1972) and visualized with PhyD3 (Kreft et al., 2017). Type-based species clustering using a 70% digital DNA-DNA hybridization radius around type strains was performed as previously described (Meier-Kolthoff and Göker, 2019). Subspecies clustering was performed using a 79% digital DNA-DNA hybridization threshold as previously described (Meier-Kolthoff et al., 2014).

For a more robust analysis based of 16S rRNA sequences, pairwise sequence similarities were calculated using the method recommended by Meier-Kolthoff et al. (2013b) for 16S rRNA genes available via the GGDC web server^4^ (Meier-Kolthoff et al., 2013a), and phylogenies were inferred (Meier-Kolthoff et al., 2013a) using the DSMZ phylogenomics pipeline (Meier-Kolthoff et al., 2014) adapted to single genes. A multiple sequence alignment was created with MUSCLE (Edgar, 2004). Maximum likelihood (ML) and maximum parsimony (MP) trees were inferred from the alignment with RAxML (Stamatakis, 2014) and TNT (Goloboff et al., 2008), respectively, using the GTR-CAT model and midpoint rooting (Hess and De Moraes Russo, 2007). For maximum likelihood, rapid bootstrapping in conjunction with the autoMRE bootstrapping criterion (Pattengale et al., 2010) and a subsequent search for the best tree was used. The maximum likelihood bootstrapping converged after 300 replications, yielding an average branch support of 72%. For maximum parsimony, 1000 bootstrapping replicates were used in conjunction with tree-bisection-and-reconnection branch swapping and ten random sequence addition replicates. The sequences were checked for compositional bias using the X^2^ test as implemented in PAUP^∗^ (Swofford, 2002). The average support derived from bootstrapping by maximum parsimony was 66%.

### Pangenome Analysis

The pangenome of all the listed strains in this study was analyzed using the Bacterial Pan-Genome Analysis (BPGA) tool (Chaudhari et al., 2016). Furthermore, the species-specific pan genomes for subset-1 [strains B (2011), B^*T*^ (2015), and D] and subset-2 (strains 2, A, E, N, S, and MS40/45) were analyzed in a similar manner. Apart from these species-specific analyses, a genus-specific pangenome using one representative of each species/subspecies (strains B^*T*^ (2015), N, MS40/45, and BV2-C) was also calculated. Venn diagrams of the pangenome matrix were prepared using R-software.

## Results and Discussion

### General Description and Properties of Nine Axenic Isolates of *Chloracidobacterium* spp.

The current type-strain, *Chloracidobacterium* sp. strain B^*T*^ (2015), was isolated from an enrichment culture of *Synechococcus* sp. Type B′, which was isolated from Octopus Spring (Tank and Bryant, 2015a). Octopus Spring is nearby and chemically similar to Mushroom Spring in the Lower Geyser Basin of YNP (Tank et al., 2017). To understand better the properties of members of the genus *Chloracidobacterium*, additional isolates were sought from two hot spring systems. Seven of the eight remaining strains isolated in this study came from mat samples collected from the main effluent channel of Mushroom Spring, YNP (see Table 1 and Supplementary Figure 1). The source pool of Mushroom Spring has a temperature of approximately 69°C, and Supplementary Figure 1 shows the approximate locations of collection sites at 40–45, 52, and 60°C. The remaining axenic strain, BV2-C, was isolated from Pool 2, sampling site C, of Baba Vanga Sanctuary at Rupite hot springs, Bulgaria. This isolate came from a submerged mat sample taken at ∼40°C in the area shown in Supplementary Figure 2. Other *Chloracidobacterium* spp. were detected in mat samples collected at various sampling sites at Rupite, but it was not possible to obtain axenic isolates.

Cells of strains B^*T*^ (2015) and D, as well as the original enrichment strain B (2011) (Bryant et al., 2007), grow in suspension in liquid culture and do not readily sediment, while strains E, N, and BV2-C grow in clumps and aggregates and readily sediment in still (unshaken) cultures (see Table 1 and Supplementary Figure 3. Strain MS40/45 exhibits an intermediate growth behavior between these two extremes (Supplementary Figure 3). The perceived color of the cultures differs as a result of the cell concentration as well as these two growth modes. When cells of strains B or D grow to high cell density in suspension, the concentrated cultures appear burnt orange in color, but at lower cell densities the cultures appear yellow-green to orange-green (Supplementary Figure 3). Cultures of the strains that grow in clumps and aggregates usually have a greenish-brown color, and they tend to appear browner as the aggregates become larger (see Supplementary Figure 3). The growth behavior in liquid culture does not correlate with the site of isolation nor the temperature. Aggregate formation could partly be an adaptation to growth in effluent channels by providing a strategy to prevent cells from being washed out by the overflowing spring water.

Although all strains were mostly rod-shaped, cell shape is generally rather pleomorphic, and considerable variation in cell length and to a lesser extent cell diameter is evident in these images (Supplementary Figures 4, 5). Strain B^*T*^ (2015) was reported to form predominantly solitary cells with dimensions of ∼2.5 × 0.8–1.0 μm (Tank and Bryant, 2015a), and strain D is very similar in appearance (Supplementary Figures 4A,B). Strains N, E, and MS40/45 produce cells that are longer (up to ∼5 μm) and slightly narrower, and they showed a strong tendency to form aggregates (Supplementary Figures 3, 4C–E). Cells of strain BV2-C are similar to those of stain B^*T*^ (2015) but are slightly longer and showed a greater tendency to aggregate (Supplementary Figure 4F).

The growth behavior of six of the strains as a function of temperature was investigated in more detail (Supplementary Figure 6). None of the axenic strains are able to grow at 60°C or higher in spite of the fact that some of the strains were isolated from mat samples collected at 60°C. Strains B^*T*^ (2015) and D are unable to grow at temperatures lower than 40°C, and optimal growth of these strains occurs at about 50°C. Strains MS40/45, E and N cannot grow at temperatures below 30°C, but all three grow better at lower temperatures than strains B^*T*^ (2015) and D. Strain E grows at slightly higher temperatures than strain N, but the optimal growth temperatures for these two strains are similar (∼50°C) and are slightly higher than that for MS40/45 (∼45°C). The growth behavior of strain BV2-C with respect to temperature is similar to that of strain MS40/45. Strain BV2-C cannot grow below 30°C nor above 55°C and exhibits optimal growth at ∼45°C. Finally, preliminary experiments showed that some of the strains might be less sensitive to oxygen than others and that this varies as a function of temperature (data not shown). However, these differences were not systematically studied.

*Chloracidobacterium thermophilum* strain B (2011) can produce four Chl pigments: Chl *a* esterified with phytadienol (Chl *a*_*PD*_), BChl *a* esterified with phytol (BChl *a*_*P*_), BChl *c* esterified with numerous alcohols, and Zn-BChl *a*′ esterified with phytol (Zn-BChl *a*_*P*_′) (Garcia Costas et al., 2011, 2012b; Tsukatani et al., 2012). Strain B (2011) also produces several carotenoids, including echinenone, canthaxanthin, lycopene, γ-carotene, and β-carotene (Garcia Costas et al., 2012b) and deoxyflexixanthin (Tsukatani et al., 2012), Strain E was previously shown to have a different distribution of BChl *c* homologs than strain B and similar carotenoids but in different amounts and ratios (Tank and Bryant, 2015b). Pigment analyses of strain BV2-C indicate that this strain also synthesizes the same major (B)Chl and carotenoid pigments as strain B^*T*^ (2015) (Supplementary Figure 7). Partial analyses of pigments in some other strains (D, E, N, MS40/45) indicated that each strain produced a somewhat different array of BChl *c* homologs but that similar carotenoids including echinenone were produced by all of the strains (data not shown).

**FIGURE 1:**
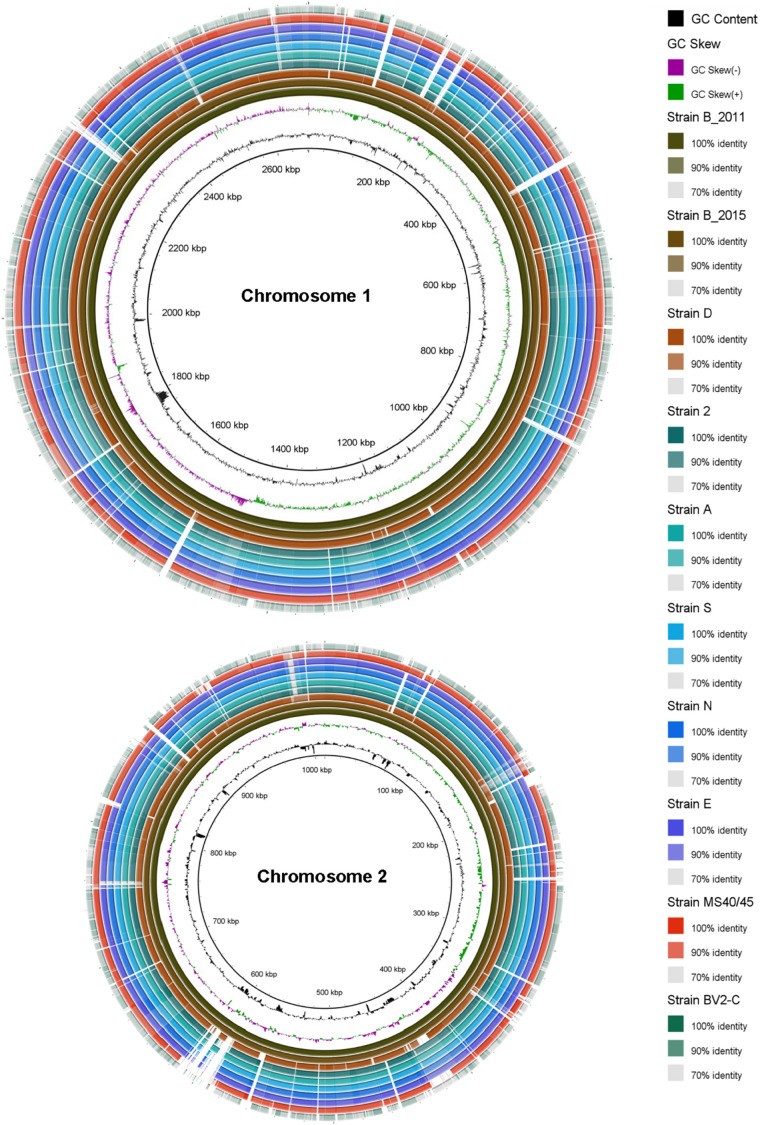
Circular comparative representation of the *Chloracidobacterium* spp. whole genomes based upon BLAST-based homology of nucleotide sequences. The complete genome of each strain comprises two chromosomes with different lengths (∼2.6 Mbp and ∼1.0 Mbp, respectively; see Table 1). The genome of strain B (2011) was used as the reference genome. Circles (from inside to outside) 1 and 2 depict GC content (black line) and GC skew (magenta and green lines); circle 3: strain B^*T*^ (2011) genome; circle 4: strain B^*T*^ (2015) genome mapped against the strain B^*T*^ (2011) genome; circle 5: strain D genome mapped against the strain B^*T*^ (2011) genome; circle 6: strain 2 genome mapped against the strain B (2011) genome; circle 7: strain A genome mapped against the strain B (2011) genome; circle 8: strain S genome mapped against the strain B (2011) genome; circle 9: strain N genome mapped against the strain B (2011) genome; circle 10: strain E genome mapped against the strain B (2011) genome; circle 11: strain MS40/45 genome mapped against the strain B (2011) genome; circle 12: strain BV2-C genome mapped against the strain B (2011) genome. Legends on right hand side showing color gradient for% similarity. BRIG 0.95 was used to build the circular representation (Alikhan et al., 2011). Mapping studies were done using BLASTn with an e-value cut-off of 1 × e^–5^.

A variety of information concerning the original enrichment culture [“strain B (2011)”] and the nine axenic isolates is summarized in Table 1. *C. thermophilum* strain B^*T*^ (2015), which was provisionally designated as the type strain (Tank and Bryant, 2015a), is an axenic isolate derived from the original enrichment culture, strain B (2011), the genome of which was determined by pyrosequencing using the 454 platform (Garcia Costas et al., 2012a). All of the other genomes were sequenced using the PacBio RSII or Sequel platforms. Strains B (2011), B^*T*^ (2015), and D have very similar mol% G + C contents of 61.34, 61.31, and 61.48, respectively. Strains 2, A, E, N, and S likewise have very similar but slightly lower mol% G + C contents of 61.13 to 61.18; strain MS40/45 has a slightly higher mol% G + C content of 62.68; and strain BV2-C has a notably lower mol% G + C content of 59.9. As first reported for the “*Ca.* C. thermophilum” genome, i.e., “strain” B (2011), the genomes of all nine axenic isolates are encoded on two circular DNA chromosomes of approximately 2.7 and 1.0 Mbp (Figure 1, Table 1). The total genome sizes for the strains are very similar, ranging from 3.635 to 3.777 Mbp. Each of the genomes encodes a single rRNA gene cluster and further encodes 46 to 49 tRNA molecules and 3161 to 3424 protein coding sequences (CDS) (Table 1).

### Genome Comparisons of *Chloracidobacterium* Isolates

The previously published genome determined for the original enrichment culture, “*Ca*. C. thermophilum strain B (2011)” (Garcia Costas et al., 2012a), was used as the basis of comparison for pair-wise comparisons of nine axenic strains. The genome of the clonal axenic strain B^*T*^ (2015) is most similar to the published sequence of the enrichment strain B (2011), but the sequence for the clonal axenic isolate is not identical to the consensus genome sequence of the non-clonal enrichment culture from which it was derived. The least similar genome is that of strain BV2-C, the isolate from Rupite hot springs in Bulgaria (Figure 1). Between these two extremes, the other isolates from Mushroom Spring are clearly more similar to “*Ca*. C. thermophilum” “strain B (2011)” (Garcia Costas et al., 2012a) and B^*T*^ (2015) from Octopus Spring than to strain BV2-C. These strains share numerous insertions/deletions (indels), although examples of strain-specific indels are also observed (see Figure 1). Although regions of the genomes showing lower average sequence identity can be identified throughout in the comparisons, the strains from Mushroom Spring generally have a much higher degree of sequence identity than strain BV2-C from Bulgaria.

**FIGURE 2:**
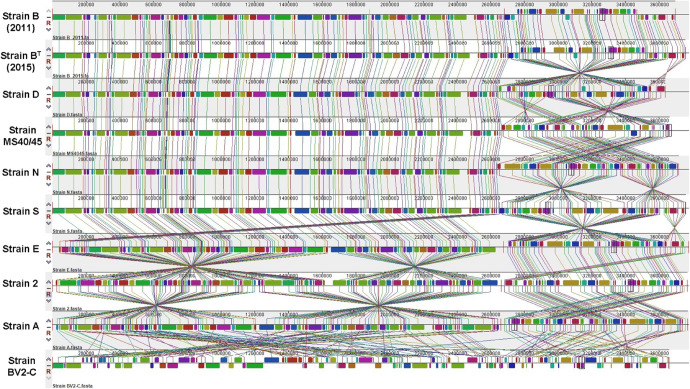
Multiple sequence alignments calculated by MAUVE. Global multiple sequence alignments of ten *Chloracidobacterium* spp. genomes to evaluate synteny, large-scale rearrangements, and inversions. Sequences from 1 to about 2.6 Mbp correspond to chromosome 1 and sequences covering the last ∼1.0 Mbp correspond to chromosome 2. A thin, vertical red line marks the junction of chromosomes 1 and 2.

A simultaneous alignment of all ten genomes by MAUVE was used to assess colinearity and genomic rearrangements (Figure 2; Darling et al., 2004). The region from 1 to about 2.6 Mbp corresponds to chromosome 1 and the remaining ∼1 Mbp to the right corresponds to chromosome 2. The genomes of strains B (2011) and B^*T*^ (2015) from Octopus Spring are nearly completely colinear except for some indels that occur at about 300 kbp in chromosome 1. The closely related strain D from Mushroom Spring (see below) is also largely colinear with B^*T*^ (2015) but chromosome 2 has two large, inverted regions. Similarly, strains MS40/45, N, and S are largely colinear with strains B and D throughout chromosome 1, but chromosome 2 in these three strains has numerous inversions that differ among these strains. The remaining three strains (strains E, 2, and A) from Mushroom Spring have large regions of both chromosomes that are colinear with the others described above, but there are multiple large inversions on both chromosomes (Figure 2). Thus, all strains from Mushroom Spring have genomes that are similar in length and organization, and that are at least locally colinear.

The obvious exception is the genome of strain BV2-C isolated from Rupite Springs in Bulgaria, which is extensively rearranged with numerous inversions and translocations on both chromosomes. Most of the genome of strain BV2-C is non-syntenous with the genomes of all strains from YNP, although the gene contents are similar (see below).

**FIGURE 3:**
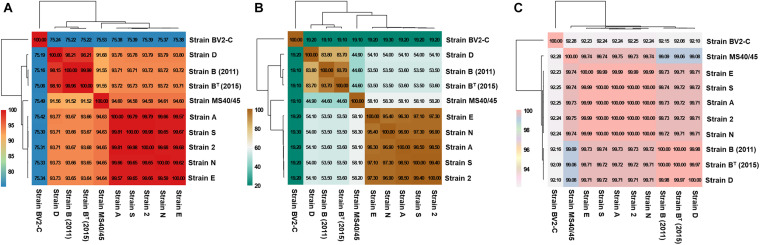
Heatmaps representing the hierarchical clustering of *Chloracidobacterium* spp. strains based on genomic distances for the evaluation of species circumscription. The extent of nucleotide identity was calculated according to different indices: **(A)** Average Nucleotide Identity calculated by BLAST (ANIb); **(B)** digital DNA-DNA hybridization (dDDH); and **(C)** tetranucleotide frequency (TETRA) as shown. Values in boxes indicates the percentage of genomic relatedness for each metric. Colors indicate strains with similar percent identity values. The heatmap was generated in R package plots using the heatmap.2 function.

Based on genomic distance calculations for the evaluation of species circumscription (Richter and Rosselió-Móra, 2009), heatmaps based on hierarchical clustering of the sequence data from the nine axenic *Chloracidobacterium* strains are shown in Figure 3. Three assessment methods clearly and consistently delineate three strain groups; one strain, MS40/45, is intermediate between two of the strain clusters. Figure 3A shows a heatmap comparison of the ten genomes based upon average nucleotide identity calculated by BLAST (ANIb), and this comparison method assigns the strains to four groups. In the first group, strains B (2011) and B^*T*^ (2015) (from Octopus Spring) are nearly identical, and an additional strain isolated from Mushroom Spring, strain D, is very similar with an ANIb score of 98%. Isolate MS40/45 is more distantly related at ∼91.5%. Strains 2, A, E, N, and S form the largest cluster of isolates, and they are very similar based on ANIb, with scores between ∼99.6 and 99.9%. Members of this cluster are slightly more closely related to MS40/45 (∼94.6%) than they are to strains B and D (∼93.7%). Strain BV2-C from Rupite hot springs is clearly distinctive by this metric and is approximately equally distant from the other nine sequences with an identity value of about 75% ANIb found for all pair-wise comparisons.

Figure 3B shows a heatmap comparison of the ten genomes based on digital DNA-DNA hybridization (dDDH; Auch et al., 2010). The data exhibit a similar pattern to those shown in Figure 3A but provide somewhat greater resolution. Strains 2, A, E, N, and S again form a well-defined cluster with pair-wise comparison values ranging from 95.4 to 99.4%. These five strains are quite well separated from strains B (2011), B^*T*^ (2015), and D (∼54%). Strain MS40/45 is again intermediate from this cluster (∼58%) and strains B and D (45%), which are themselves much more similar (∼84%). Strain BV2-C from Rupite hot springs is again only distantly related to the other sequences, exhibiting a very low dDDH value of ∼19%. This lower value is expected in part from the lower mol% G + C content of that genome (Table 1).

Figure 3C shows results based on a comparison of tetranucleotide frequencies for the ten sequences (TETRA; Teeling et al., 2004; Richter et al., 2016). Although numerically this approach may appear less significant, in fact it produces results in complete agreement with the methods just described. Because it is often used as a parameter to inform the binning of sequences in metagenomic analyses, the small but highly consistent differences seen in Figure 3C illustrates how binning based upon oligonucleotide frequency may sometimes lead to situations in which sequences from closely similar but discrete species could be grouped together in a single bin.

### 16S rRNA Sequence Comparisons

**FIGURE 4:**
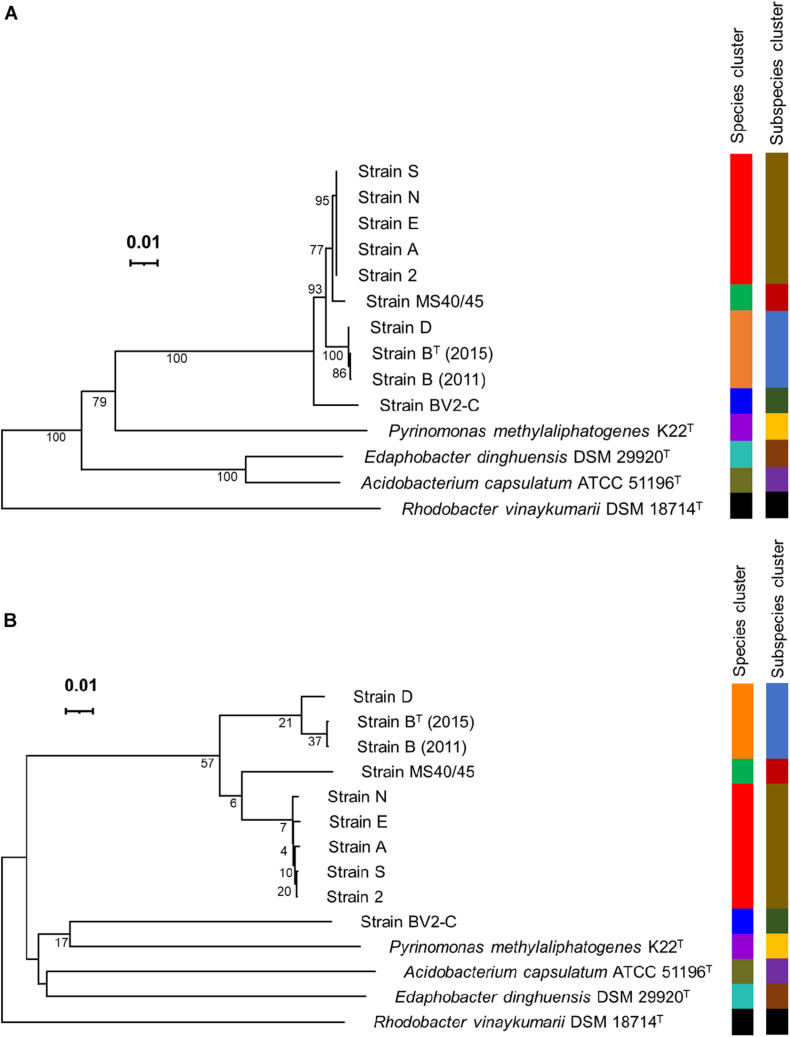
Phylogenetic trees based on 16S rRNA sequences and whole-genome sequences. **(A)** Phylogenetic tree showing the position of *Chloracidobacterium* spp. strains based upon the 16S rRNA gene. **(B)** Phylogenetic tree based upon Genome BLAST Distance Phylogeny (GBDP) distances for complete genomes. The trees were constructed using FastME v.2.1.6.1 software, which calculates 16S rRNA and GBDP distances; the branch lengths are scaled in terms of GBDP distance formula *d*_5_. The numbers above the branches are GBDP pseudo-bootstrap support values, with average branch support of 85.5 and 21.7% for the 16S rRNA gene and complete genome trees, respectively. The trees are rooted at the midpoint. Leaf labels are color-coded by affiliation to species and subspecies clusters. The results were obtained using the Type Strain Genome Server (TYGS), a free bioinformatics platform available at https://tygs.dsmz.de.

Pairwise sequence identity values for comparisons of the 16S rRNA sequences of the ten sequences are shown in Table 2. The sequences for the enrichment culture [B (2011)] from Octopus Spring and the axenic strain derived from it [B^*T*^ (2015)] are identical, and that of a very closely related strain from Mushroom Spring, strain D, is 99.93% identical to those two sequences. Strains 2, A, E, N, and S, have identical 16S rRNA sequences, which are about 99% identical to those of strains B and D. The 16S rRNA sequence of strain MS40/45 was 98.4% identical to the sequences for strains B and D and 99.4% identical to the 16S rRNA sequences of the other strains from Mushroom Spring. Pairwise comparisons of the 16S rRNA sequence from strain BV2-C from Rupite hot springs showed identities ranging from ∼97% for strains B and D to ∼98% identity to strains 2, A, E, N, and S from Mushroom Spring. The 16S rRNA sequence comparisons are depicted in the phylogenetic tree shown in Figure 4A. The 16S rRNA sequences for all *Chloracidobacterium* spp. genomes form a well-supported clade that is most closely related to *Pyrionomonas methylaliphatogenes* (Crowe et al., 2014). Within the *Chloracidobacterium* clade, strain BV2-C is the earliest diverging member followed by strains B and D and then all of the remaining strains from Mushroom Spring (strains MS40/45, 2, A, E, N, and S).

**TABLE 2:**
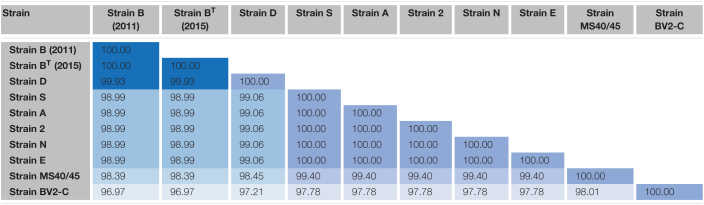
Pairwise percent similarity values for 16S rRNA sequences of strains of *Chloracidobacterium* spp.

Figure 4B shows a phylogenetic tree based upon the whole genomes for the same strains as described above for Figure 4A based upon 16S rRNA sequences. This tree is based on Genome Blast Distance Phylogeny, a method that is based upon dDDH (see Figure 3B and the preceding section; Ahn et al., 2017; Meier-Kolthoff and Göker, 2019). This tree is nearly identical to that for 16S rRNA with one notable exception. Strain BV2-C, which surprisingly has a low dDDH percentage score compared to all other *Chloracidobacterium* spp. sequences, grouped together with *P. methylaliphatogenes* K22 (Crowe et al., 2014) rather than forming the earliest diverging lineage of the *Chloracidobacterium* clade in this analysis. Considering all results presented here, this must be an artifact due to long-branch attraction, but it does emphasize how different strain BV2-C from Rupite hot springs is from the geographically isolated strains from YNP.

A second phylogenetic tree based upon 16S rRNA sequences, which includes a much broader range of strains belonging to the phylum *Acidobacteriota*, is shown in Supplementary Figure 8. This tree shows the relationships among family level clades of strains, and it clearly shows that all *Chloracidobacterium* spp. isolates described here form a monophyletic clade that is distinct from all other most closely related families (e.g., *Blastocatellaceae*, *Arenimicrobiaceae*, and *Pyrinomonadaceae*) within the phylum *Acidobacteriota*. These differences, together with the strong similarities in physiology (e.g., chlorophototrophy) and metabolism (e.g., inability to synthesize branched-chain amino acids), are defining. Thus, we will propose the creation of a new family, *Chloracidobacteriaceae*, within the phylum *Acidobacteriota*, class *Blastocatellia*, order *Blastocatellales*. A formal proposal and description of the family and its members will be published elsewhere.

### Speciation

The data described herein support the assignment of the nine currently available axenic isolates to at least three *Chloracidobacterium* species, and thus we tentatively propose the following. Firstly, *Chloracidobacterium thermophilum* strains B^*T*^ (2015) and D, are moderately thermophilic organisms (growth at temperatures between 40 and 60°C) that grow in suspension culture without aggregation forming yellowish-green to burnt orange, non-settling cultures (Tank and Bryant, 2015a, b). Secondly, *Chloracidobacterium aggregatum* strains 2, A, E, N, S, and MS40/45 are mesophilic to moderately thermophilic organisms (optimum growth from 45 to 55°C) that grow in greenish-brown clumps and aggregates in liquid culture. Thirdly, *Chloracidobacterium validum* strain BV2-C is a moderately thermophilic species that grows in clumps and aggregates at temperatures of 30 to 55°C. Strains B^*T*^ (2015) and D are similar to the originally described *Chloracidobacterium thermophilum* enrichment culture strain B (2011) (Bryant et al., 2007), and examples of this species have been isolated from both Octopus Spring [strains B (2011) and B^*T*^ (2015)] and Mushroom Spring (strain D). All *C. aggregatum* strains described herein were isolated from Mushroom Spring mats at different positions (and temperatures) in the effluent channel (Table 1), which correspond to temperatures ranging from 40 to 60°C. Thus, these strains likely represent different ecotypes of *C. aggregatum*. Strain BV2-C of *C. validum* was isolated from a geographically distant location compared to the other strains, and this is likely to be a contributing factor in its distinctive properties.

Strain MS40/45 is a borderline case, and it is uncertain whether it should be assigned as an ecotypic variant of *C. aggregatum* or possibly a different species. Strain MS40/45 has slightly higher mol% G + C content, a slightly smaller genome, and dDDH values that suggest it could possibly be a different species (“*C. fuscum*”). However, the 16S rRNA sequence of this strain is nearly identical to those of strains 2, A, E, N, and S (99.4%), and its genome is mostly syntenic with the genomes of *C. aggregatum* strains S and N. Consistent with its clumpy growth behavior in liquid, there is presently insufficient evidence to designate this strain as another species. Thus, we have designated this isolate as an ecotype of *C. aggregatum* at this time (also see results below from pangenome analysis that are consistent with this decision).

*Chloracidobacterium validum* has clearly diverged substantially from all of the isolates from YNP, and future studies will attempt to establish how this isolate differs from *C. thermophilum* and *C. aggregatum* metabolically and physiologically. Because of the rules of the Bacteriological Code, we plan to propose *C. validum* as the type species for the genus *Chloracidobacterium*, which would become the type species of a to be proposed new family, *Chloracidobacteriaceae*.

### Genome Content

**FIGURE 5:**
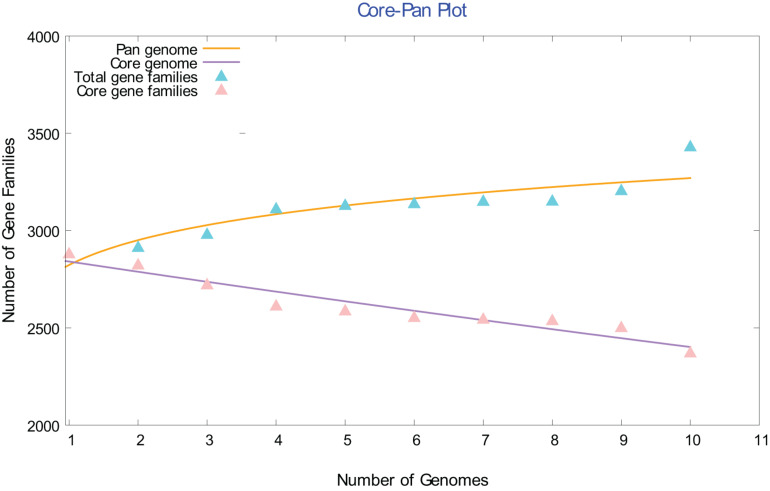
Comparison of the core and pangenome of the genus *Chloracidobacterium*. The open pangenome character of the *Chloracidobacterium* spp. strain genomes is illustrated by this core *versus* pangenome plot. As the number of included genomes increases, the pangenome increases and the conserved core gene families decreases.

A full description of the gene contents of these ten genomes is beyond the scope of this study. However, a few broad, general observations can be mentioned here. To gain insights into species evolution, a pan-genome analysis of *Chloracidobacterium* spp. was performed using the Bacterial Pan-Genome Analysis (BPGA) tool. Bacterial Pan-Genome Analysis uses USEARCH to cluster orthologous proteins to determine the core (conserved), accessory (dispensable), and unique (strain-specific) gene-pools of a species (Chaudhari et al., 2016). The core genome of the genus *Chloracidobacterium* spp. increased, and the number of core gene families decreased slowly, as the number of genomes included in the analysis increased from one to ten (Figure 5). This pattern suggests that the genus *Chloracidobacterium* has an open pangenome, which is likely to be impacted by the acquisition of new genes and functions by horizontal gene transfer and which could impact speciation. This point is evident in the defining property of the group, namely chlorophototrophy. It seems highly likely that ancestors of *Chloracidobacterium* spp. acquired the capacity for (B)Chl biosynthesis, as well as chlorosomes, FMO, and type-1 reaction centers, by lateral gene transfer from a member(s) of the phyla *Chlorobiota* and/or *Chloroflexota* (Bryant et al., 2012; Bryant and Liu, 2013). A comparison of the ten genome sequences showed that the genus *Chloracidobacterium* contains ∼2373 core genes that are found in all members of fully sequenced genomes (Supplementary Table 2). A second group of “accessory genes,” comprising 268 to 530 genes, was found in most but not all of the other genomes. Each genome contains a few (e.g., one gene in strain N) to many (e.g., 225 genes in strain BV2-C) unique genes that are not found in any other genome. Correspondingly, each genome is missing a few genes (one to as many as 130 genes) compared to the published genome sequence of the enrichment culture [“strain B (2011)”].

Venn diagrams from the pangenome analysis for *C. thermophilum*, which includes strains B (2011), B^*T*^ (2015), and D, show that these three strains have a pangenome of 2978 genes (Supplementary Figure 9A) *C. thermophilum* had 2735 core genes, and most of the accessory genes were shared between the genomes of strains B^*T*^ (2015) and strain B (2011). This is reasonable because strain B^*T*^ (2015) is a clonal isolate derived from the enrichment here denoted strain B (2011). Strain D had the largest number of strain-specific genes (62), which could partly reflect that this strain was isolated from Mushroom Spring while the B strains were isolated from Octopus Spring. This finding shows that strains of the same species having similar genomic features and phylogenetic position nevertheless exhibit some differences in their gene contents, which may be due to their different geographic isolation and/or to the slight physico-chemical differences in these two similar but nevertheless distinctive hot springs.

*Chloracidobacterium aggregatum*, which includes strains 2, A, E, N, S, and MS40/45, had a slightly larger pangenome of 3060 genes with a core gene pool of 2647 genes (Supplementary Figure 9B). Relatively few unique genes are found in the strain-specific pool except for the genome of strain MS40/45, which has 84 unique genes. This finding confirms that strains 2, A, E, N, and S are likely to be ecotypes of a single species. The possible exception is strain MS40/45, which may be a more divergent ecotype or possibly a different species, because it has the largest number of unique genes. To determine whether MS40/45 represents new species or a more divergent ecotype of *C. aggregatum*, we performed a pangenome analysis including representatives of each species of the genus *Chloracidobacterium* and strain MS40/45. If the number of unique genes in MS40/45 is greater than 84 in the pangenome of genus *Chloracidobacterium* in comparison to the pangenome of *C. aggregatum*, then it should probably be considered a new species, but if not, then it seems more likely to be an ecotype of *C. aggregatum*.

**FIGURE 6:**
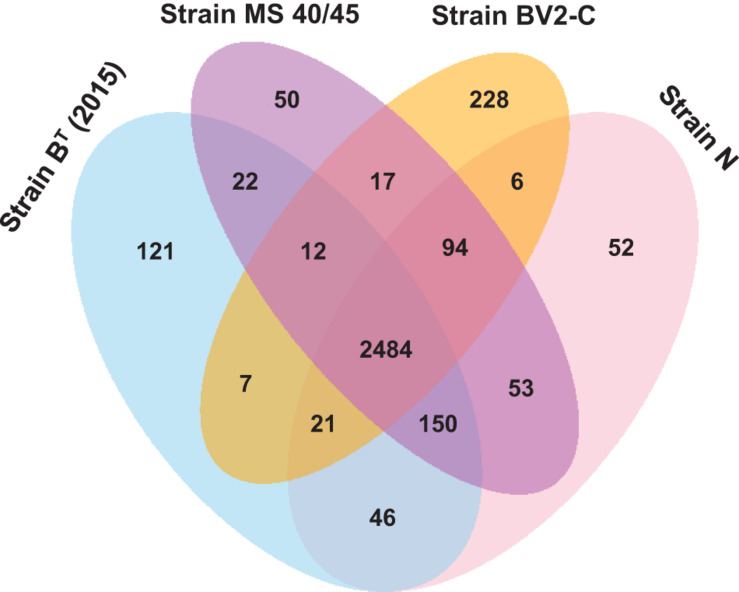
Venn diagram showing the distribution of core, accessory and unique genes shared between the genomes of *C. thermophilum* strain B^*T*^ (2015), *C. aggregatum* strain N, *C. aggregatum* strain MS40/45, and *C. validum* strain BV2-C. This analysis includes most of the genes found in the pangenome for all *Chloracidobacterium* spp. strains described in this study.

The Venn diagram in Figure 6 shows that strains B^*T*^ (2015), N, MS40/45, and BV2-C from the *Chloracidobacterium* genus have the largest pangenome with 3364 genes. However, despite having the largest pangenome, the four included strains [strains B^*T*^ (2015), N, BV2-C, and MS40**/**45**]** have the smallest core gene pool of 2484 genes. The largest number of accessory genes (150) are shared between strains B_2015, N and MS40/45, perhaps because these three strains were all isolated from hot springs in the Lower Geyser Basin of YNP. Strain BV2-C, which is geographically isolated (Bulgaria) from all other strains analyzed (YNP), has a larger number of unique gene (228) compared to other three strains (only 50 to 121). This finding is consistent with the finding that strain BV2-C is most diverged compared to the other *Chloracidobacterium* spp. strains as described above. Finally, strain MS40/45 has the smallest number of unique genes (50) in this genus-specific pangenome analysis, while in the species-specific pangenome of *C. aggregatum* this strain had 84 unique genes. This smaller number of unique genes (50 *vs.* 84) suggests that this strain should probably be considered an ecotype of *C. aggregatum* rather than as a new species in this genus, which might be expected to have more unique genes—even though a single gene could confer the capacity to occupy a different mat niche. Further genomic analyses and physiological studies will be required to determine the taxonomic position of this strain.

The pangenome for all *Chloracidobacterium* spp. strains was further analyzed to check the distribution of core, accessory, and unique genes relative to KEGG metabolic pathways (Supplementary Figure 10). The absence of unique genes in important categories, such as amino acid metabolism and energy metabolism, indicates that all strains have similar metabolic pathways for these essential processes. The genomes of all the strains were found to be similar in their functional profiling to one another and exhibited remarkable similarity to those of the genome of strain B (2011) (Garcia Costas et al., 2012a). All genomes encoded the enzymes necessary to make heme *a*, heme *b*, Chl *a*, BChl *a*, and BChl *c*, as well as those required to make the carotenoids echinenone, lycopene, γ-carotene and β-carotene. Genes encoding the type-1 reaction center *(pscA, pscB*), the BChl *a*-binding FMO protein (*fmoA*), and chlorosome envelope (e.g., *csmA*) were present in all genomes. This is logical because all strains are obligate photoheterotrophs as reported for *C. thermophilum* (Tank and Bryant, 2015a, b). Interestingly, all *Chloracidobacterium* spp. lack the ability to synthesize branched chain amino acids, but all have an ABC transporter for taking up branched chain amino acids, and all have enzymes for their degradation. Similarly, all strains appear to be unable to synthesize lysine and vitamin B_12_, although some enzymes required for terminal steps in the vitamin B_12_ pathway are present; this suggests that strains can probably salvage this valuable resource from their environments. Finally, and perhaps most surprisingly, all strains characteristically synthesize tyrosine using phenylalanine 4-monooxygenase and dioxygen and can synthesize Chls using both AcsF and oxygen or BchE and water. These observations are consistent with the fact that all strains require oxygen but are microaerophiles that are unable to grow on air and grow best at about 1% (v/v) oxygen.

## Conclusion

*Chloracidobacterium* spp. stand out among members of the phylum *Acidobacteriota* for several reasons. Firstly, they uniquely (so far) have the capacity to use light as an energy source, using multiple types of Chls, several similar carotenoids, type-1 reaction centers, and chlorosomes as light-harvesting antenna complexes. Secondly, they are unusual because conditions have been found to grow and isolate these organisms axenically from multiple complex communities. Thirdly, *Chloracidobacterium* spp. are moderate thermophiles, although there is evidence that a third species occurs in Mushroom Spring mats at temperatures as high as 68°C (David M Ward and Jason Wood, personal communication). Fourthly and perhaps most surprisingly, none of the isolates to date can synthesize branched chain amino acids, but all isolates can take up and degrade branched chain amino acids. It will be fascinating to learn more about the ecophysiology of these most unusual members of the phylum *Acidobacteriota*.

## Data Availability Statement

The datasets presented in this study can be found in online repositories. The names of the repository/repositories and accession number(s) can be found below: NCBI (accession: PRJNA717397).

## Author Contributions

MS, MT, and DB planned and directed the research and participated in data analyses. MT, MS, and YS isolated the new strains reported. MT supervised MS and YS during their studies. MS performed all comparative genomic analyses reported here. AG isolated the original enrichment culture of *C. thermophilum* strain B devoid of *Synechococcus* type B′ and sequenced its genome. NS performed the pigment analyses. VT studied the growth modes of some of the *C. aggregatum* isolates. DD-M and SS sequenced and assembled the genomes of six isolates. AS and IA assembled the genomic sequence data for four strains. SHr and SHn advised MS on technical issues. SHn provided funding for studies conducted in Tokyo. DB provided funding for strain isolation, DNA sequencing, and the original studies leading to isolation and characterization of *C. thermophilum*. MS and DB wrote the manuscript, and all other authors contributed according to their various skills and knowledge.

## Conflict of Interest

Authors VT and MT are employed by DSMZ – German Culture Collection of Microorganisms and Cell Cultures, GmbH. The remaining authors declare that the research was conducted in the absence of any commercial or financial relationships that could be construed as a potential conflict of interest.

## Footnotes

1 https://github.com/PacificBiosciences/smrtmake

2 https://www.ezbiocloud.net/

3 https://tygs.dsmz.de

4 http://ggdc.dsmz.de/

## Supplementary Material

Supplementary Figure 1 Effluent channel of Mushroom Spring, in the Lower Geyser Basin of Yellowstone National Park, WY, United States. The approximate temperatures of the source pool and sampling sites in the channel are indicated on the figure. The lush growth of the microbial mat community, dominated by *Synechococcus* spp. and *Roseiflexus* spp. (Klatt et al., 2011), is clearly visible in the channel.

Supplementary Figure 2 Marcus Tank at the sampling site at Pool 2, sampling site C, of Baba Vanga Sanctuary at Rupite Hot Springs, Rupite, Bulgaria. The approximate temperature (40°C) at the site is indicated. The bottle on the right shows a cyanobacterial enrichment culture from which *Chloracidobacterium validum* strain BV2-C was isolated.

Supplementary Figure 3 Appearance of cultures of strains of *Chloracidobacterium* spp. strains in liquid culture. Liquid cultures of *C. thermophilum* strain B^*T*^ (2015), *C. aggregatum* strains D, N, E, and MS40/45, and *C. validum* strain BV2-C.

Supplementary Figure 4 Phase-contrast micrographs of strains of *Chloracidobacterium* spp. The panels show **(A)** *C. thermophilum* strain B^*T*^ (2015); **(B)** *C. aggregatum* strain D; **(C)** *C. aggregatum* strain N; **(D)** *C. aggregatum* strain E; **(E)** *C. aggregatum* strain MS40/45/; and **(F)** *C. validum* strain BV2-C. The size bar indicates 15 μm.

Supplementary Figure 5 Fluorescence micrographs showing fluorescence from BChl *c* for various strains of *Chloracidobacterium* spp. The panels show **(A)** *C. thermophilum* strain B^*T*^ (2015); **(B)** *C. aggregatum* strain D; **(C)** *C. aggregatum* strain N; **(D)** *C. aggregatum* strain E; **(E)** *C. aggregatum* strain MS40/45/; and **(F)** *C. validum* strain BV2-C.

Supplementary Figure 6 Growth behavior of selected *Chloracidobacterium* spp. strains as a function of temperature between 25 and 60°C. The panels show data for *C. thermophilum* strain B^*T*^ (2015); *C. aggregatum* strain D; *C. validum* strain BV2-C; and *C. aggregatum* strains E, N, and MS40/45.

Supplementary Figure 7 HPLC analyses of pigments in *Chloracidobacterium* spp. strains. **(A)** Elution profile of monitored at 770 nm of pigment extracts from *Chloracidobacterium thermophilum* strain B^*T*^ (2015) (black line) and *Chloracidobacterium validum* strain BV2-C (red line). The peak eluting at 42.5 min represents BChl *a*. **(B)** Elution profile of monitored at 667 nm of pigment extracts from *Chloracidobacterium thermophilum* strain B^*T*^ (2015) (black line) and *Chloracidobacterium validum* strain BV2-C (red line). All peaks have the absorbance spectrum characteristic of BChl *c*. **(C)** Elution profile of monitored at 491 nm of pigment extracts from *Chloracidobacterium thermophilum* strain B^*T*^ (2015) (black line) and *Chloracidobacterium validum* strain BV2-C (red line). The identities of numbered peaks are shown in the inset. **(D)** and **(E)** In-line absorbance spectra of the numbered peaks for *Chloracidobacterium thermophilum* strain B^*T*^ (2015) **(D)** and *Chloracidobacterium validum* strain BV2-C **(E)**. The dotted green line shows the absorbance spectrum of Chl *a* from *Synechococcus* sp. PCC 7002. All peaks numbered in panel B have the absorbance spectrum of BChl *c*. Peaks eluting at different times either have different esterifying alcohols or carry different numbers of methyl groups on the side chains at C8 and/or C12 (see Garcia Costas et al., 2012b; Tank and Bryant, 2015b). The peak patterns are clearly very similar, but the amounts of individual BChl *c* homologs are very different for the two isolates. Similarly, in the carotenoid profiles in the 491-nm panel, the individual carotenoids in the two isolates are essentially identical, but the amounts of the various carotenoids are different in the two strains. However, echinenone and γ-carotene are the most abundant carotenoids in both strains.

Supplementary Figure 8 Inferred maximum likelihood phylogenetic tree based upon 16S rRNA sequences showing the relationship of *Chloracidobacterium* spp. strains to a broad range of type species from the indicated families within the phylum *Acidobacteriota*. The branches are scaled in terms of the expected number of substitutions per site. The numbers above the branches are support values when larger than 60% from maximum likelihood analysis (left) and maximum parsimony (right) based upon 1000 bootstrapping replicates. This tree provides strong support for the creation of a new family, *Chloracidobacteriaceae*, within the phylum *Acidobacteriota* (see text for additional details). The colored bars on the right demarcate strains belonging families of *Acidobacteriota* as color-coded on the left. The orange bar indicates strains in this study that belong to a novel, monophyletic family, *Chloracidobacteriaceae*.

Supplementary Figure 9 Venn diagrams showing the distribution of core, accessory and unique genes shared between the genomes *C. thermophilum* strains **(A)** and the genomes of *C. aggregatum* strains **(B)**.

Supplementary Figure 10 Percentage distribution of genes according to protein categories in the Kyoto Encyclopedia of Genes and Genomes (KEGG). The distribution of gene categories according to core (green bars), accessory (red bars), and unique proteins (blue bars) are shown. The graph was created with BPGA software (Chaudhari et al., 2016).

Supplementary Table 1 National Center for Biotechnology Information (NCBI) accession number(s) assigned to each genome and its chromosomes.

Supplementary Table 2 Pangenome analysis of *Chloracidobacterium* spp. strains showing the genome-wide numbers of core, accessory, unique, and exclusively missing genes.
