# Supplementary material for: Genomic and Phenotypic Characterization of Chloracidobacterium Isolates Provides Evidence for Multiple Species
Source: Front Microbiol. 2021 Jun 17;12:704168. doi: 10.3389/fmicb.2021.704168 (PMC8245765; doi:10.3389/fmicb.2021.704168)
Supplement: Supplementary Figure 1 — Effluent channel of Mushroom Spring, in the Lower Geyser Basin of Yellowstone National Park, WY, United States. The approximate temperatures of the source pool and sampling sites in the channel are indicated on the figure. The lush growth of the microbial mat community, dominated by Synechococcus spp. and Roseiflexus spp. (Klatt et al., 2011), is clearly visible in the channel. [file Data_Sheet_1.pdf]

Supplementary Figures 1-10 and Supplementary Tables 1 and 2

Genomic and phenotypic characterization of *Chloracidobacterium* isolates provides evidence for multiple species

Mohit Kumar Saini[1], Aswathy Sebastian[2], Yoshiki Shirotori[1], Nathan T. Soulier[3], Amaya M. Garcia Costas[3,4], Daniela I. Drautz-Moses[5], Stephan C. Schuster[5], Istvan Albert[2,3], Shin Haruta[1], Satoshi Hanada[1], Vera Thiel[1,3,6], Marcus Tank[1,3,6], Donald A. Bryant[3]*

[1]Department of Biological Sciences, Tokyo Metropolitan University, 1-1 Minami-Osawa, Hachioji, Tokyo 1920397, Japan; [2]The Huck Institutes for the Life Sciences, The Pennsylvania State University, University Park, PA 16802, USA; [3]Department of Biochemistry and Molecular Biology, The Pennsylvania State University, PA 16802, USA; [4]Department of Biology, Colorado State University-Pueblo, Pueblo, CO 81001, USA; [5]Singapore Centre for Environmental Life Sciences Engineering, Nanyang Technological University, Singapore; [6]DSMZ – German Culture Collection of Microorganisms and Cell Cultures, GmbH Inhoffenstraße 7B 38124 Braunschweig, Germany

***Author for correspondence: Donald A. Bryant**, Dept. of Biochemistry and Molecular Biology, The Pennsylvania State University, S-002 Frear Laboratory, University Park, PA 16802; Telephone: 814-865-1992; Fax: 814-863-7024; E-mail: dab14@psu.edu

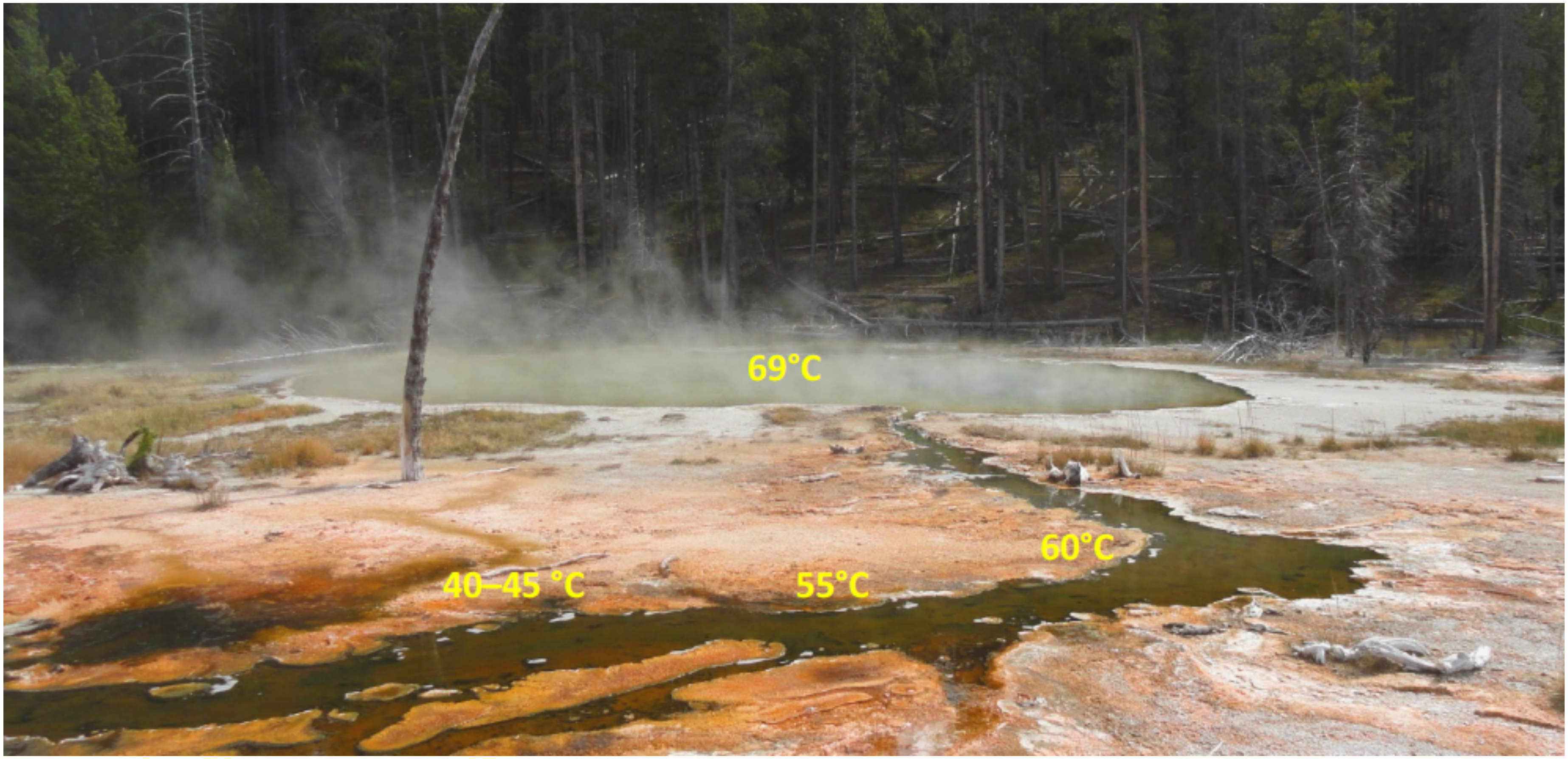

SUPPLEMENTARY FIGURE 1. Effluent channel of Mushroom Spring, in the Lower Geyser Basin of Yellowstone National Park, WY, USA. The approximate temperatures of the source pool and sampling sites in the channel are indicated on the figure. The lush growth of the microbial mat community, dominated by *Synechococcus* spp. and *Roseiflexus* spp. (Klatt et al. 2012), is clearly visible in the channel.

Pool 2, Site C, Baba Vanga Sanctuary at Rupite Hot Springs, Bulgaria

Enrichment

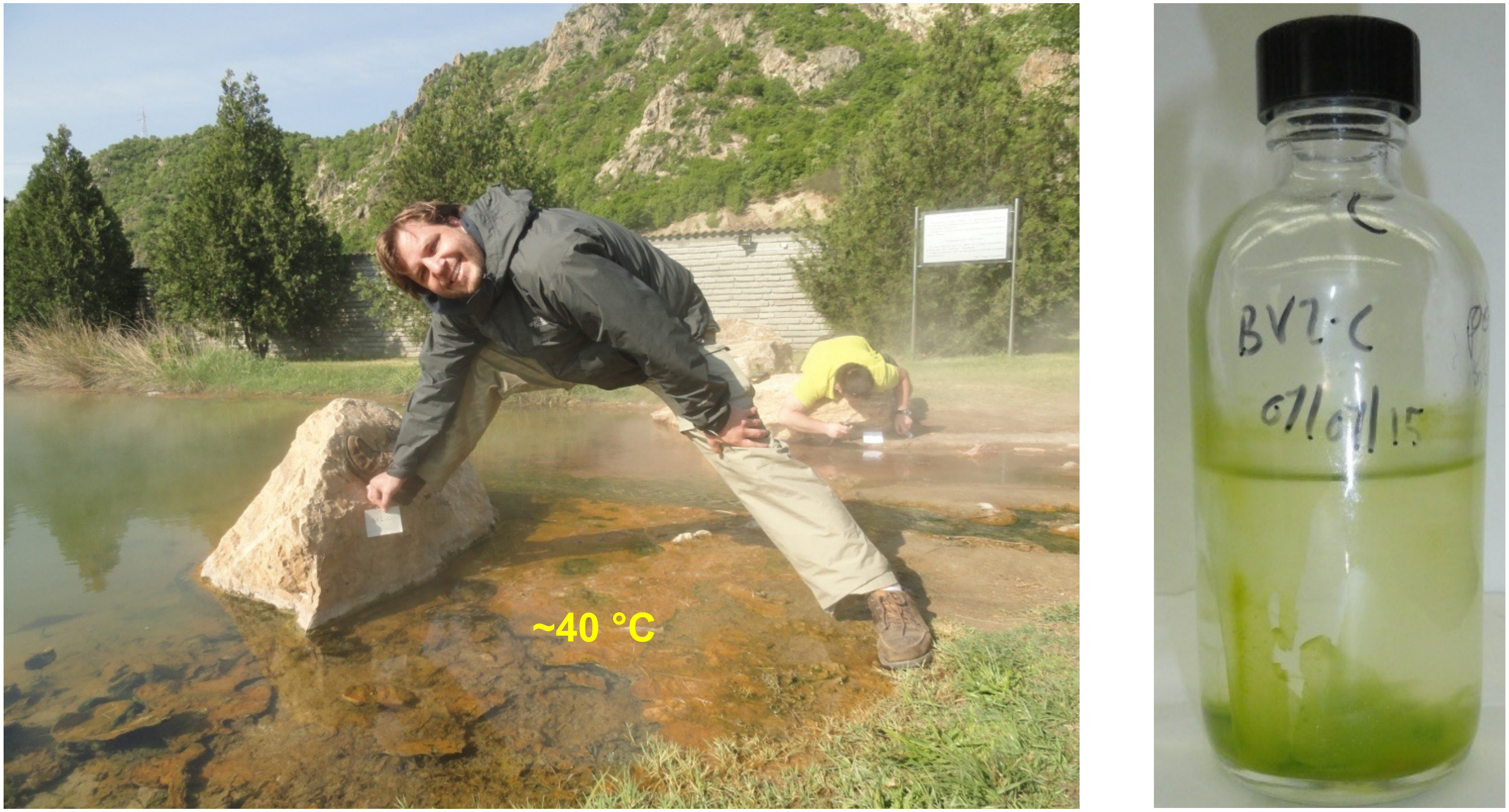

SUPPLEMENTARY FIGURE 2. Dr. Marcus Tank at the sampling site at Pool 2, sampling site C, of Baba Vanga Sanctuary at Rupite Hot Springs, Rupite, Bulgaria. The approximate temperature (40°C) at the site is indicated. The bottle on the right shows a cyanobacterial enrichment culture from which *Chloracidobacterium validum* strain BV2-C was isolated.

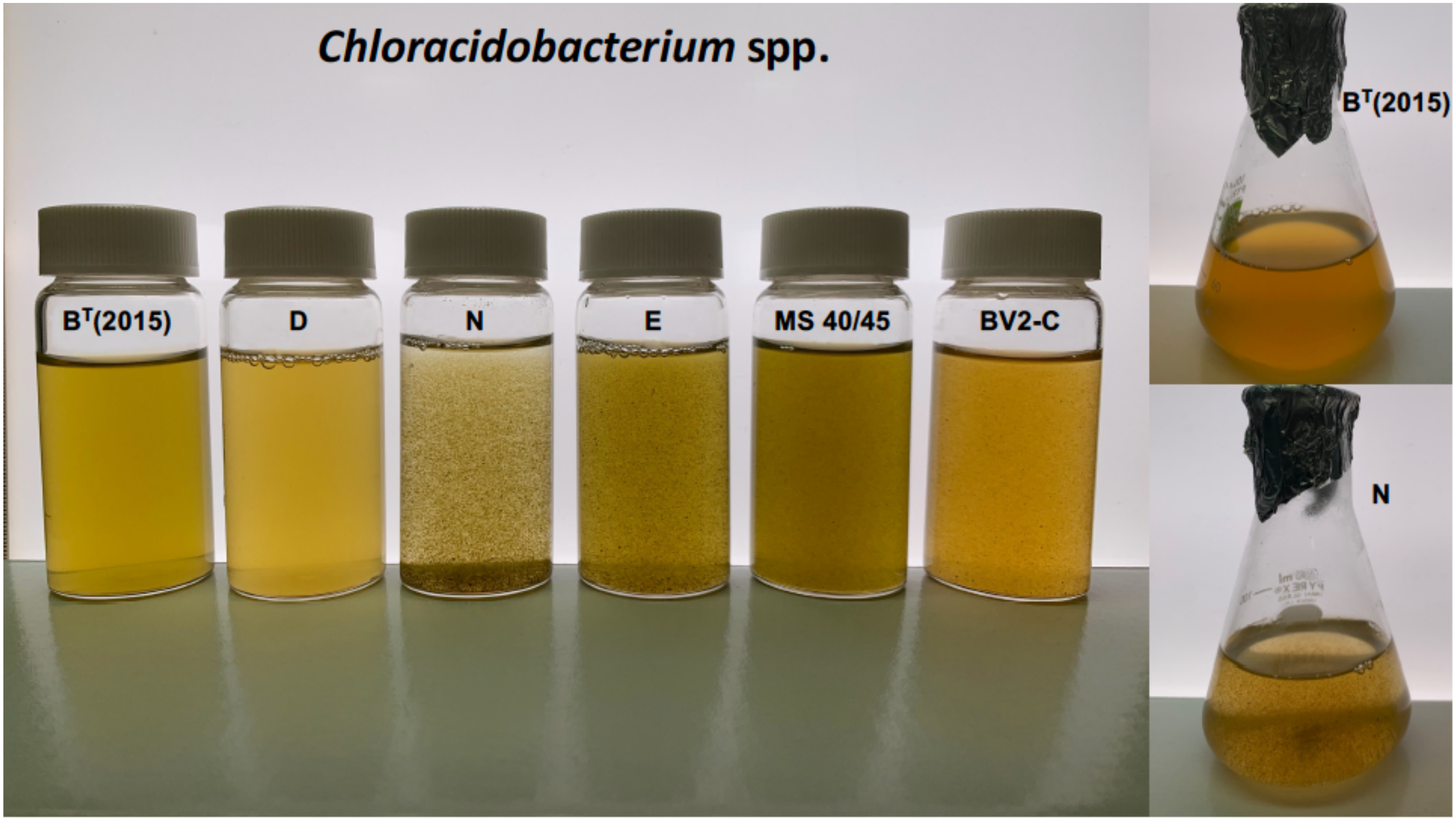

SUPPLEMENTARY FIGURE 3. Appearance of cultures of *Chloracidobacterium* spp. strains in liquid culture. Liquid cultures of *C. thermophilum* strain B^T (2015); *C. thermophilum* strain D; *C. aggregatum* strains N, E, and MS40/45; and *C. validum* strain BV2-C.

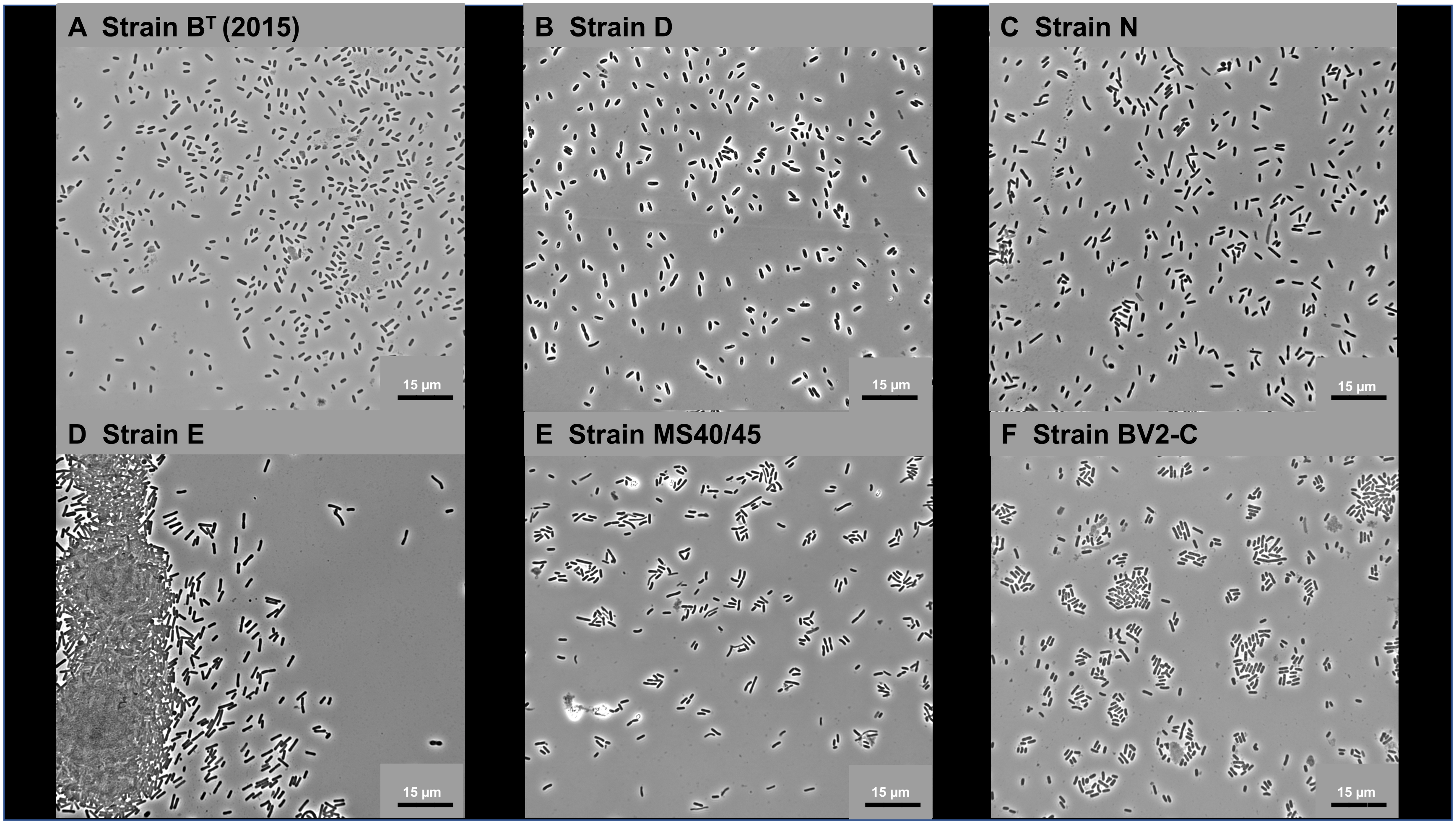

SUPPLEMENTARY FIGURE 4. Phase-contrast micrographs of strains of *Chloracidobacterium* spp. The panels show (**A**) *C. thermophilum* strain B^T (2015); (**B**) *C. thermophilum* strain D; (**C**) *C. aggregatum* strain N; (**D**) *C. aggregatum* strain E; (**E**) *C. aggregatum* strain MS40/45/; and (**F**) *C. validum* strain BV2-C. The size bar indicates 15 µm.

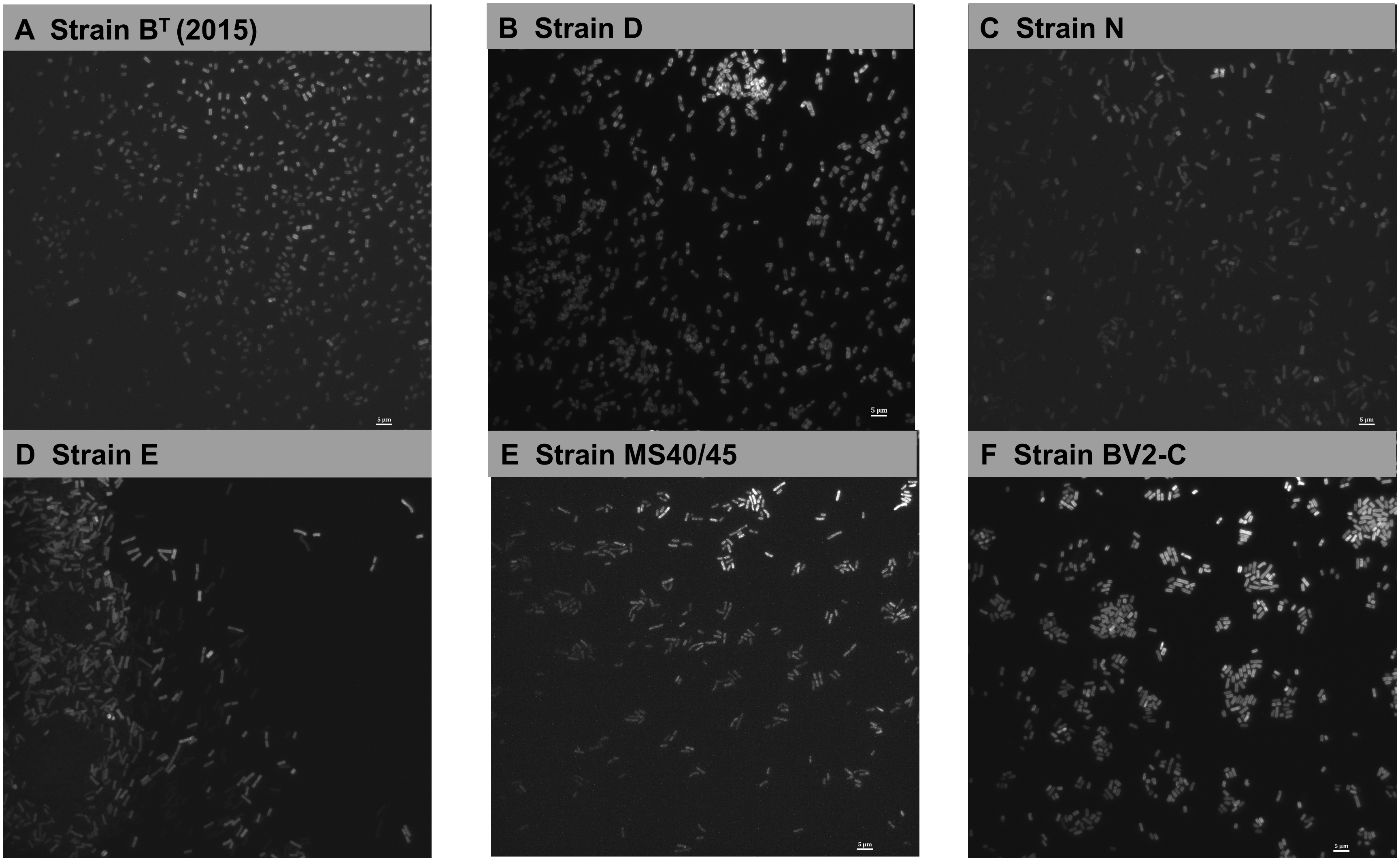

SUPPLEMENTARY FIGURE 5. Fluorescence micrographs showing fluorescence from BChl *c* for various strains of *Chloracidobacterium* spp. The panels show (**A**) *C. thermophilum* strain B^T (2015); (**B**) *C. thermophilum* strain D; (**C**) *C. aggregatum* strain N; (**D**) *C. aggregatum* strain E; (**E**) *C. aggregatum* strain MS40/45; and (**F**) *C. validum* strain BV2-C.

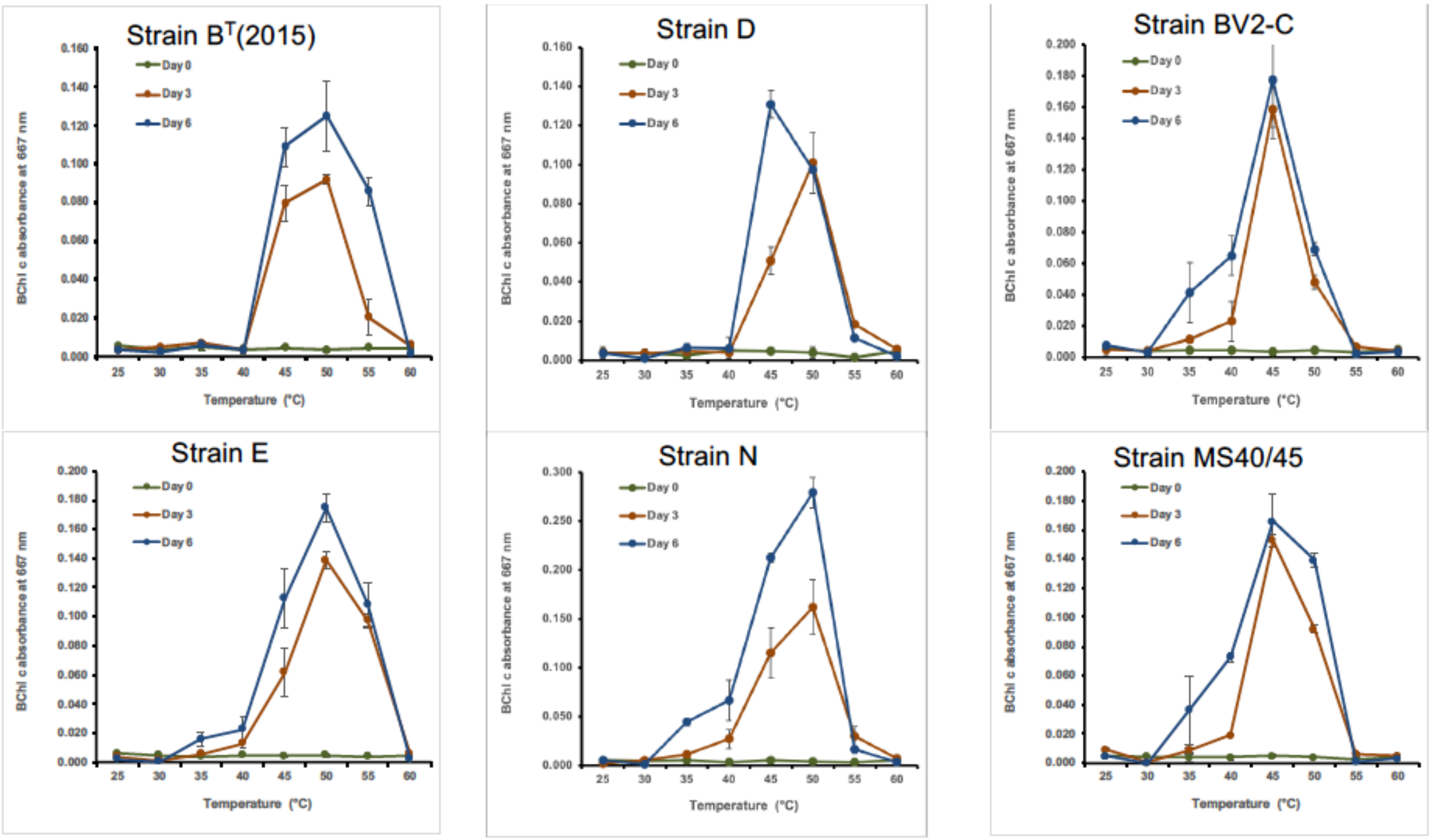

SUPPLEMENTARY FIGURE 6. Growth behavior of selected *Chloracidobacterium* strains as a function of temperature between 25 and 60°C. The panels show data for *C. thermophilum* strain B^T (2015); *C. thermophilum* strain D; and *C. validum* strain BV2-C; and *C. aggregatum* strains E, N, and MS40/45. Each strain was grown in duplicate cultures, and the average values and standard deviations are plotted.

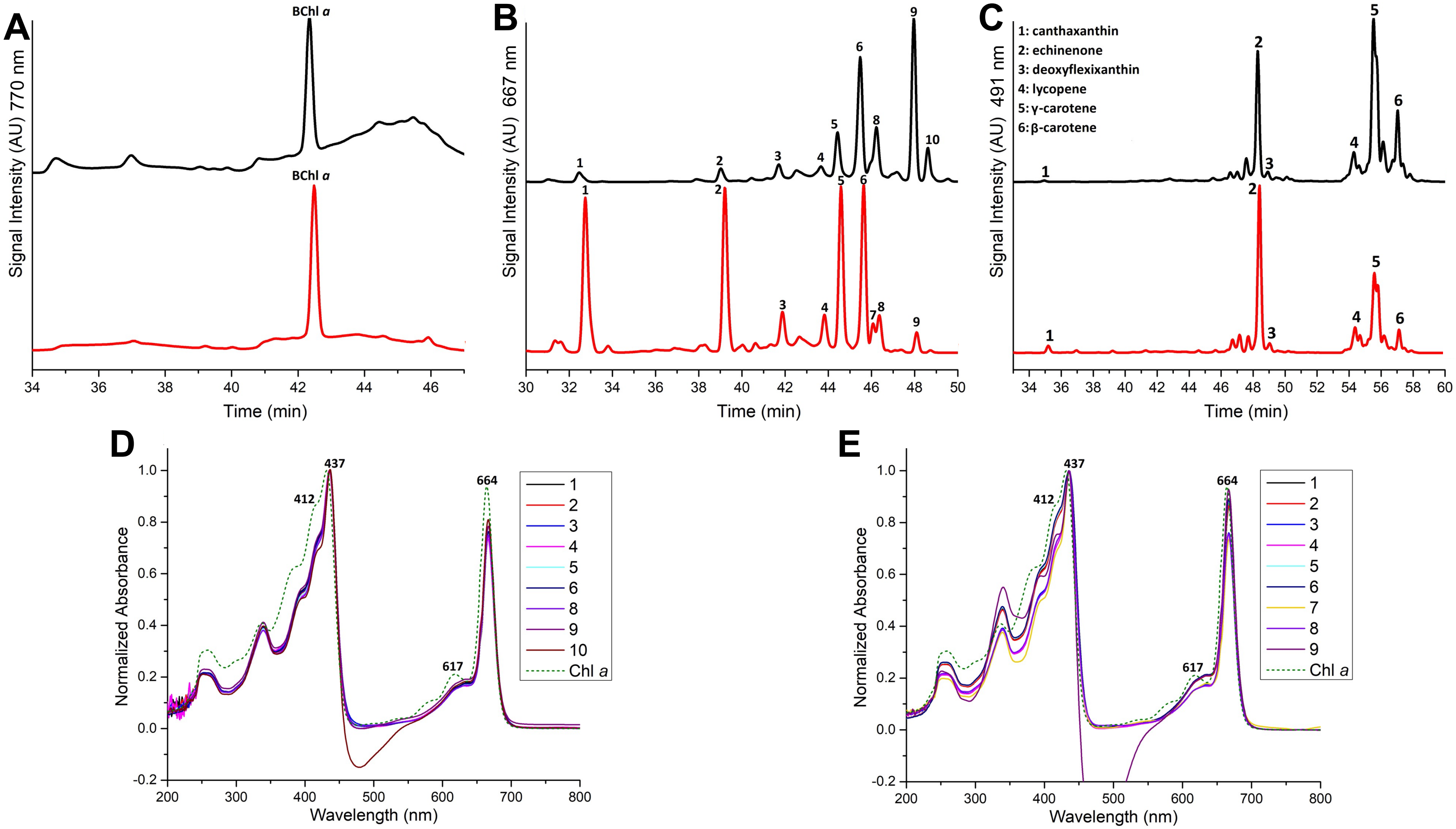

SUPPLEMENTARY FIGURE 7. HPLC analyses of pigments in *Chloracidobacterium* spp. strains. (A) Elution profile of monitored at 770 nm of pigment extracts from *C. thermophilum* strain B^{T} (2015) (black line) and *C. validum* strain BV2-C (red line). The peak eluting at 42.5 minutes represents BChl *a*. **(B)** Elution profile of monitored at 667 nm of pigment extracts

from *C. thermophilum* strain B^T (2015) (black line) and *C. validum* strain BV2-C (red line). All peaks have the absorbance spectrum characteristic of BChl *c*. (**C**) Elution profile of monitored at 491 nm of pigment extracts from *C. thermophilum* strain B^T (2015) (black line) and *C. validum* strain BV2-C (red line). The identities of numbered peaks are shown in the inset. **(D)** and **(E)** In-line absorbance spectra of the numbered peaks for *C. thermophilum* strain B^T (2015) **(D)** and *C. validum* strain BV2-C **(E)**. The dotted green line shows the absorbance spectrum of Chl *a* from *Synechococcus* sp. PCC 7002. All peaks numbered in panel B have the absorbance spectrum of BChl *c*. Peaks eluting at different times either have different esterifying alcohols or carry different numbers of methyl groups on the side chains at C8 and/or C12 (see Garcia Costas et al. 2012b; Tank et al. 2015b). The peak patterns are clearly very similar, but the amounts of individual BChl *c* homologs are very different for the two isolates. Similarly, in the carotenoid profiles in the 491-nm panel, the individual carotenoids in the two isolates are essentially identical, but the amounts of the various carotenoids are different in the two strains. However, echinenone and γ-carotene are the most abundant carotenoids in both strains.

SUPPLEMENTARY FIGURE 8. Inferred maximum likelihood phylogenetic tree based upon 16S rRNA sequences showing the relationship of *Chloracidobacterium* spp. strains to a broad range of type species from the indicated families within the phylum *Acidobacteriota*. The branches are scaled in terms of the expected number of substitutions per site. The numbers above the branches are support values when larger than 60% from maximum likelihood analysis (left) and maximum parsimony (right) based upon 1000 bootstrapping replicates. This tree provides strong support for the creation of a new family, *Chloracidobacteriaceae*, within the phylum *Acidobacteriota* (see text for additional details). The colored bars on the right demarcate strains belonging families of *Acidobacteriota* as color-coded on the left. The orange bar indicates strains in this study that belong to a novel, monophyletic family, *Chloracidobacteriaceae*.

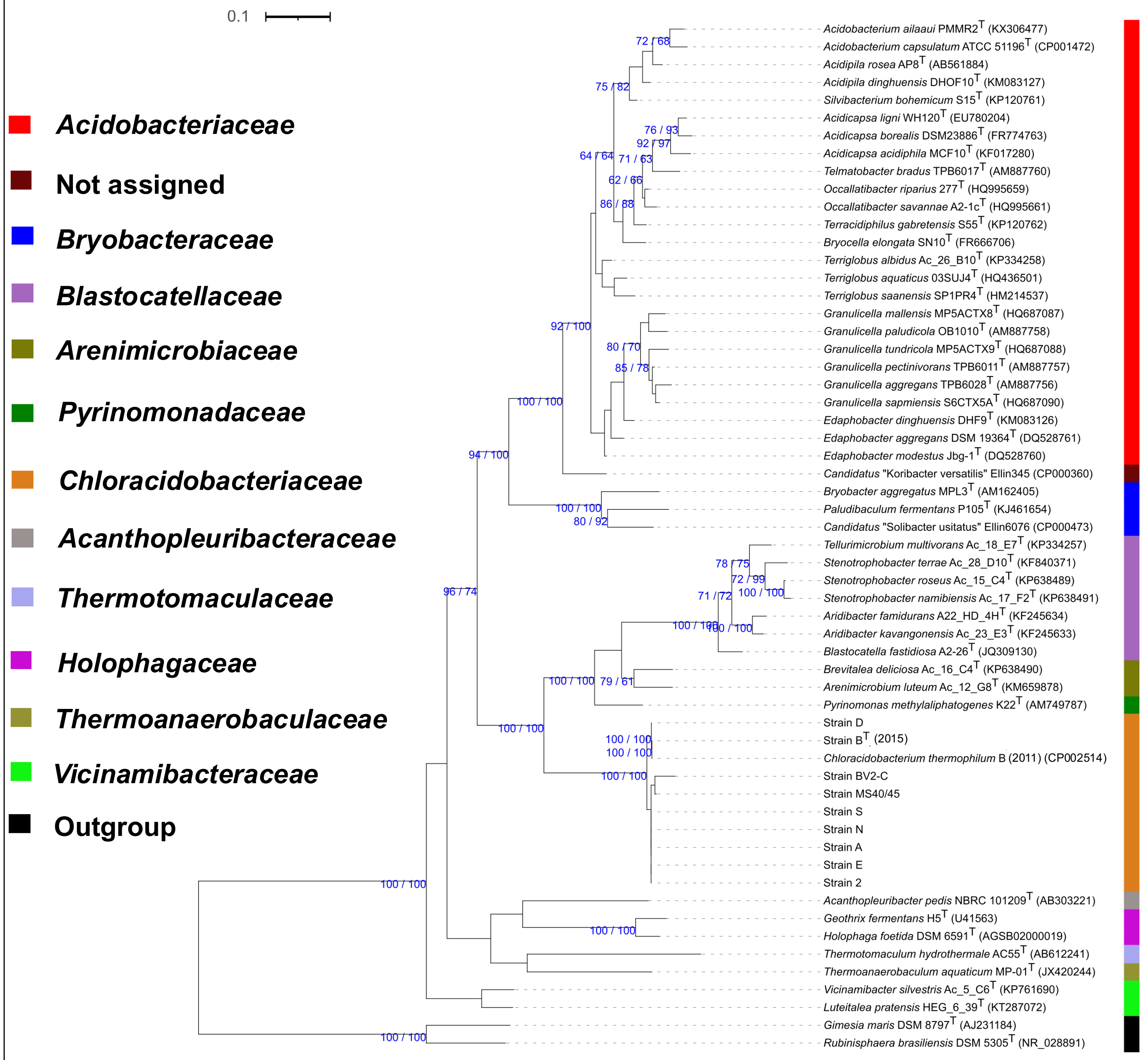

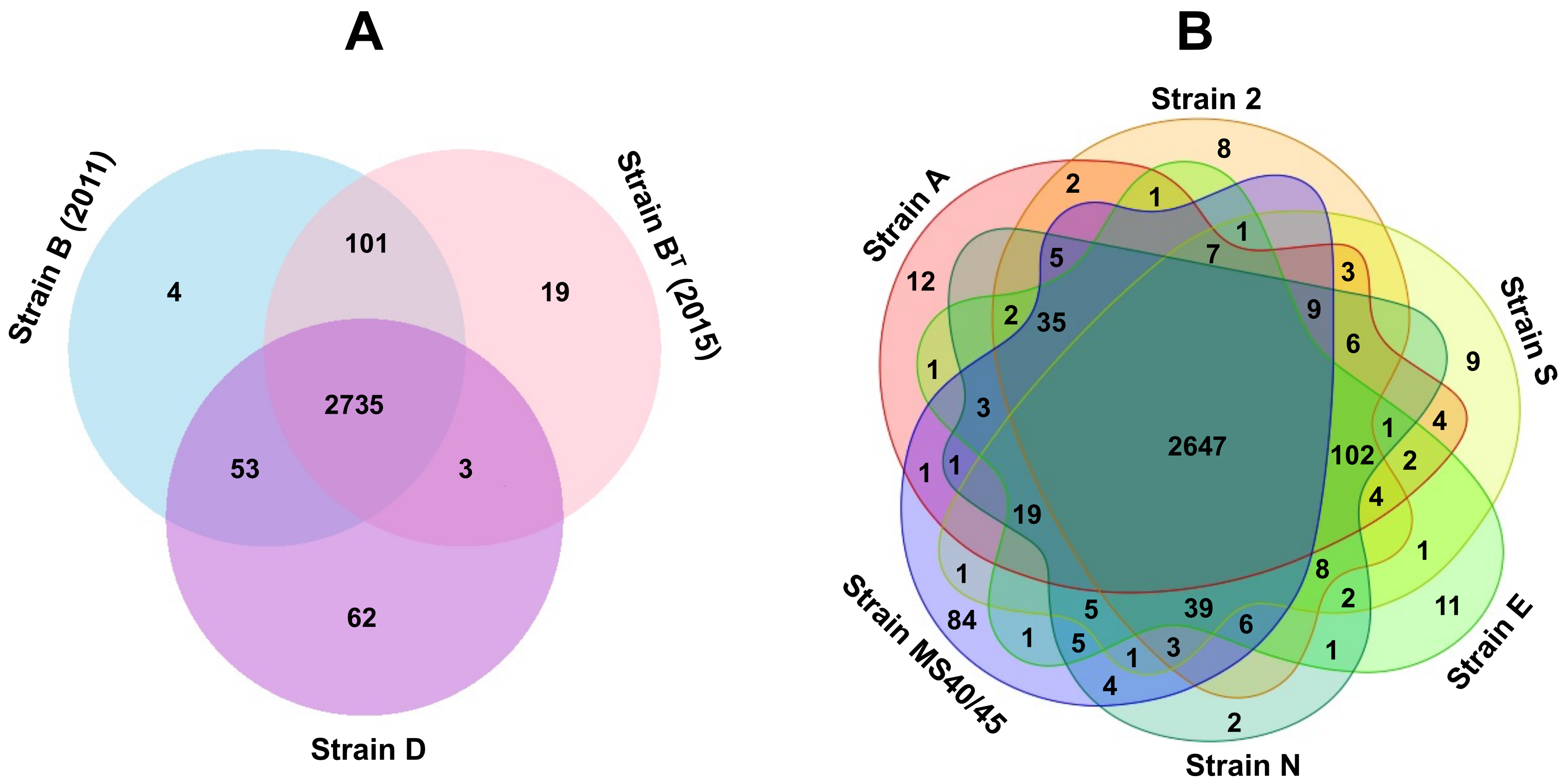

SUPPLEMENTARY FIGURE 9. Venn diagrams showing the distribution of core, accessory and unique genes shared between the genomes *C. thermophilum* strains (A) and the genomes of *C. aggregatum* strains (B).

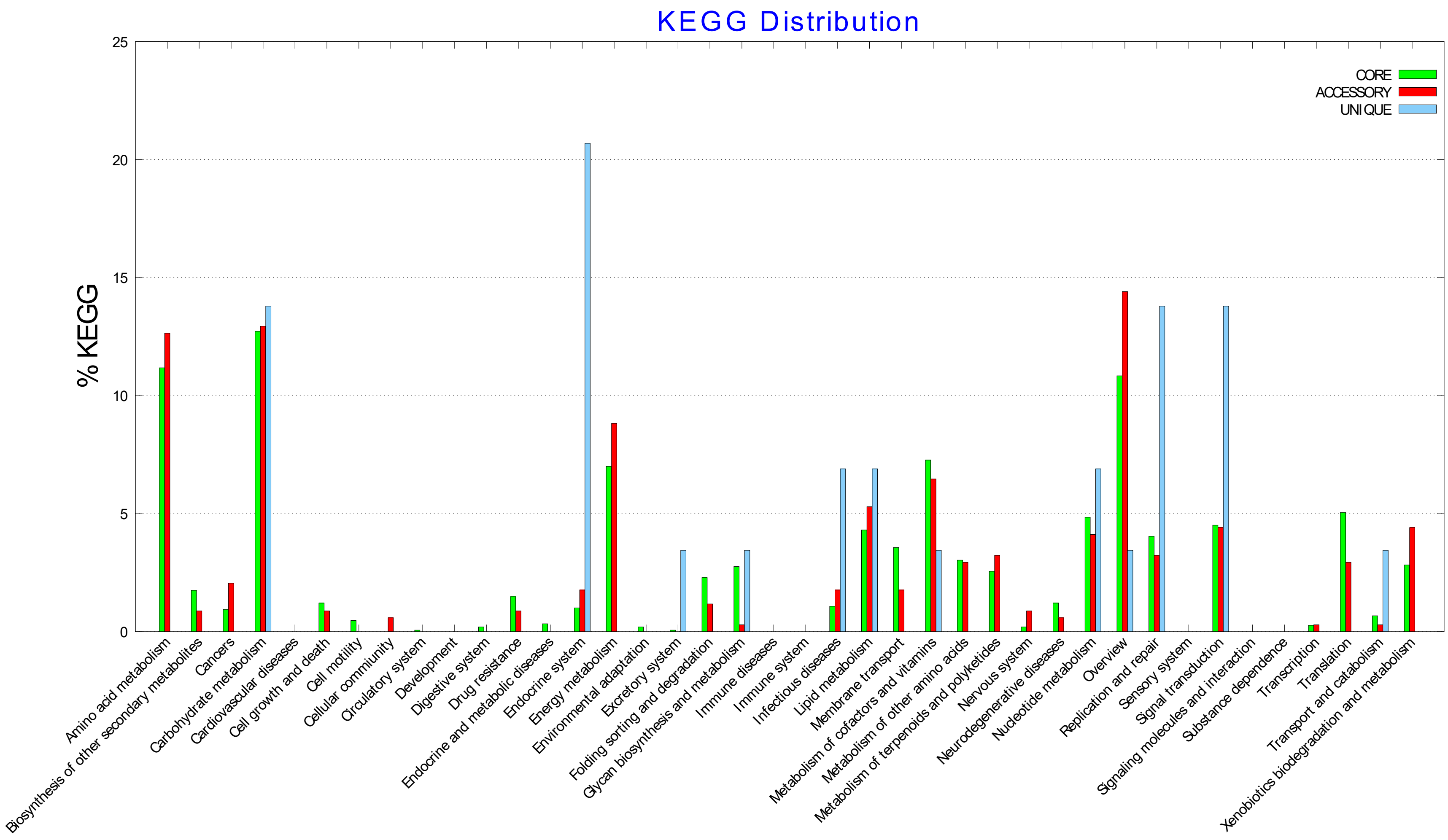

SUPPLEMENTARY FIGURE 10. Percentage distribution of genes according to protein categories in the Kyoto Encyclopedia of Genes and Genomes (KEGG). The distribution of gene categories according to core (green bars), accessory (red bars), and unique proteins (blue bars) are shown. The graph was created with BPGA software (Chaudhari et al. 2016).

SUPPLEMENTARY TABLE 1. NCBI accession number(s) assigned to each genome.

Strain	BioProject	BioSample	Accession number		Reference
			Chromosome 1	Chromosome 2	
**Strain B (2011)**	PRJNA62457	SAMN02603950	CP002514	CP002515	Garcia Costas et al. 2012a
**Strain B^T (2015)**	PRJNA717397	SAMN18499028	CP072632	CP072633	This study
**Strain D**	PRJNA717397	SAMN18499029	CP072634	CP072635	This study
**Strain 2**	PRJNA717397	SAMN18499030	CP072636	CP072637	This study
**Strain A**	PRJNA717397	SAMN18499032	CP072640	CP072641	This study
**Strain S**	PRJNA717397	SAMN18499031	CP072638	CP072639	This study
**Strain N**	PRJNA717397	SAMN18499033	CP072642	CP072643	This study
**Strain E**	PRJNA717397	SAMN18499034	CP072644	CP072645	This study
**Strain MS40/45**	PRJNA717397	SAMN18499035	CP072646	CP072647	This study
**Strain BV2-C**	PRJNA717397	SAMN18499036	CP072648	CP072649	This study

SUPPLEMENTARY TABLE 2. Pangenome analysis of *Chloracidobacterium* spp. strains showing the genome-wide numbers of core, accessory, unique and exclusively missing genes.

Strain	Core genes	Accessory genes	Unique genes	Exclusively missing genes
Strain B (2011)	2373	506	3	1
Strain B^T (2015)	2373	464	21	37
Strain 2	2373	510	7	16
Strain A	2373	486	9	26
Strain D	2373	455	18	5
Strain E	2373	523	10	6
Strain N	2373	530	1	6
Strain S	2373	497	7	27
Strain MS 40/45	2373	464	42	13
Strain BV2-C	2373	268	225	130
